# Insights on the systematics and morphology of Humiriaceae (Malpighiales): androecial and extrafloral nectary variation, two new combinations, and a new *Sacoglottis* from Guyana

**DOI:** 10.3897/phytokeys.124.34679

**Published:** 2019-06-20

**Authors:** Kenneth J. Wurdack, Charles E. Zartman

**Affiliations:** 1 Department of Botany, MRC-166, National Museum of Natural History, Smithsonian Institution, P.O. Box 37012, Washington, DC 20013-7012, USA National Museum of Natural History, Smithsonian Institution Washington D.C. United States of America; 2 Department of Biodiversity, National Institute for Amazonian Research (INPA), Av. André, Araújo 2936, Aleixo, Manaus, Amazonas 69060-001, Brazil National Institute of Amazonian Research (INPA) and Smithsonian Tropical Research Institute Manaus Brazil

**Keywords:** anthers, *Duckesia*, extrafloral nectaries, floral morphology, glands, *Humiriastrum*, *Vantanea*

## Abstract

Humiriaceae have had little recent comparative morphological study except for their distinctive fruits. We surveyed the diversity of stamen structures in the family with consideration of dehiscence patterns and the evolutionary transitions between tetra- and disporangiate anthers. Novel interpretations of floral morphology support new combinations (*Duckesialiesneri* K.Wurdack & C.E.Zartman, **comb. nov.** and *Vantaneaspiritu-sancti* K.Wurdack & C.E.Zartman, **comb. nov.**) for two species formerly in *Humiriastrum*. We investigated all eleven species of *Sacoglottis* for diagnostic features that may contribute to better species delimitations, and describe *Sacoglottisperryi* K.Wurdack & C.E.Zartman, **sp. nov.** as an endemic of the Pakaraima Mountains in western Guyana. Finally, our survey across Humiriaceae for extrafloral nectaries (EFNs) revealed their presence on leaves of all extant species as adaxial basilaminar and/or abaxial embedded glands, in addition to the frequent occurrence of marginal glandular setae. The significance of inter-generic variation in gland position and anther morphology within the family are discussed.

## Introduction

Humiriaceae contains approximately 65 species of extant trees and shrubs characterized by small white to greenish-cream (rarely red to pink) flowers and with a center of taxonomic richness in the Rio Negro Basin, the largest tributary of the Amazon River. The family is notable for its rich fossil record of distinctive, woody endocarps, and for its unusual biogeography as a nearly wholly neotropical family except for one species in tropical West Africa. The most comprehensive synoptic revision of the family or any of its eight constituent genera was published in 1961 by noted authority José Cuatrecasas. In that visionary work, Cuatrecasas radically restructured Humiriaceae with 24 new taxa, 33 new combinations, and the segregation of four new genera of which three (*Duckesia* [Ducke] Cuatr., *Hylocarpa* [Ducke] Cuatr., and *Endopleura* [Huber] Cuatr.) were monotypic Amazonian endemics. The genera as defined by Cuatrecasas (1961) are well delimited by clear discontinuities in floral bauplan, anther structure, endocarp structure, and/or placentation. In the subsequent half century advances in Humiriaceae systematics and morphology have been modest, including descriptions of 15 new species in four genera, floral anatomy (Narayana and Rao 1969–1977), palynology (Bove and Melhem 2000), and reconsideration of endocarp variation (Herrera et al. 2010, 2014). Due to the highly plastic leaf morphologies and complex infraspecific taxonomy (Cuatrecasas 1961, Cuatrecasas and Huber 1999), most species are poorly understood and research remains hampered by incomplete specimens that lack critical characters from both flowers and fruits.

Although Humiriaceae is clearly monophyletic and placed in Malpighiales, its relationships within the order (Wurdack and Davis 2009, Xi et al. 2012) and among constituent genera (Herrera et al. 2010, Bardon 2015) remain equivocal. The most robust, albeit still weak, ordinal placement to date by Xi et al. (2012) indicates Humiriaceae as sister to a parietal placentation clade (i.e., six families including Passifloraceae, Salicaceae, and Violaceae); however, this affiliation is not yet corroborated by compelling morphological evidence (see Endress et al. 2013). Divergence dating indicates it is among the oldest families of Malpighiales with a mean stem-group age of 105.7 Ma (110.0–101.6, 95% HPD) but with a relatively young crown-group age of 20.7 Ma (32.1–10.4, 95% HPD). Recent reevaluation of the fossil record provides evidence for an older crown-group diversification with at least three genera (*Duckesia*, *Sacoglottis* Mart., *Vantanea* Aubl.) by the early Oligocene (~30–28 Ma; Herrera et al. 2010, 2014).

Glandular tissues on leaves, sepals, floral disc, and/or anthers have been reported for Humiriaceae (Cuatrecasas 1961). However, foliar glands that may function as extrafloral nectaries (EFNs), in particular, have been rarely noted (i.e., Metcalfe and Chalk 1950, Belin-Depoux 1993). Cuatrecasas (1961: 88) observed that *Humiria* J. St.-Hil. leaves are “dotted with glands near the margin on the lower side” but he provided few comments for the other taxa. This lack of basic knowledge is illustrated, for example, in the online World Checklist of Extrafloral Nectaries that only lists two genera (*Sacoglottis*, *Vantanea*) and three species with EFNs for the family (Weber et al. 2015). EFNs are abundant in other families of Malpighiales, especially Euphorbiaceaeand Passifloraceae, although their distribution is likely also underreported across the order despite accounting for 26% of all EFN records in a recent tally (Weber and Keeler 2013). The adaptive value of EFNs for promoting mutualistic interactions with insects, especially ants, is well established (see Rico-Gray and Oliveira 2007). EFN ecology usually involves nectar secretion to attract ants that act as defenders to provide plant protection from herbivores.

*Sacoglottis*, the systematics focus of this study, is distinguished among the eight genera in having the lowest stamen number (10). Morphological and molecular evidence indicates a nested phylogenetic placement for *Sacoglottis* within the family (Herrera et al. 2010, Xi et al. 2012, Bardon 2015, Zartman et al., unpublished data), and consequently its androecium likely evolved via stamen reduction from higher numbers (mostly 20–30, but 100–200+ in some *Vantanea*). In its current circumscription (sensu Cuatrecasas 1961) the genus contains 10 species of trees distributed from Nicaragua to Brazil and Bolivia, as well as West African *S.gabonensis* (Baill.) Urb. A heretofore undescribed species of *Sacoglottis* with subglobose fruits has been known for many years among variously misidentified specimens from the northern Pakaraima Mountains of Guyana. While the earliest of the 20 known collections was made in 1951 by Bassett Maguire of The New York Botanical Garden, most are from later fieldwork sponsored by the Smithsonian Institution’s Biodiversity of the Guiana Shield Program, including the 2012 Kamakusa Expedition (K. Wurdack, participant; Wurdack et al. 2013) which explicitly searched for and found the species.

We herein describe this new species of *Sacoglottis* and present comparisons of its vegetative and reproductive characters in relation to the often poorly known other species in the genus. We surveyed stamen structures from exemplars of all genera to provide more detailed information on features of systematics and evolutionary interest, especially relating to anther morphology and interpretation of sporangial reductions. As a result of this objective we also present new combinations based on novel interpretations of previously overlooked floral structure for two taxa. Finally, on noting the frequent occurrence of EFNs on Humiriaceae but little mention of them in the literature, we conducted a comprehensive EFN survey of all species to document their occurrence and any qualitative generic-level distinctions.

## Materials and methods

Scanning electron microscopy (SEM) of untreated or rehydrated-ethanol transitioned then critical point dried (CPD) herbarium fragments was conducted with a Zeiss EVO MA15 (Carl Zeiss SMT, Inc., Peabody, Massachusetts) SEM at 5–10 kV after sputter coating with 10 nm of C + Au/Pd using a Leica EM ACE600 (Leica Microsystems GmbH, Wetzlar, Germany). Light microscopy (LM) was with an Olympus DSX100 (Olympus Corp., Tokyo, Japan) and extended focus imaging (EFI) for higher magnifications, or with a Zeiss Universal Compound Microscope. Androecial diversity of the family was broadly surveyed at US and then examined in detail with SEM and/or LM for representatives for each genus, including: *Duckesialiesneri* comb. nov., *Henderson 933* (US); *D.verrucosa* (Ducke) Cuatrec., *Ducke 2108* (US); *Ducke [MG-16325*] (US). *Endopleurauchi* (Huber) Cuatrec., *Baker 58* (US), *Assunção 605* (US). Humiriabalsamiferavar.imbaimadaiensis Cuatrec., *Wurdack 4814* (US); *H.crassifolia* Mart., *Cowan & Soderstrom 2145* (US). *Humiriastrumcuspidatum* (Benth.) Cuatrec., *Cid et al. 4264* (US), *Ducke 243* (US); *H.dentatum* (Casar.) Cuatrec., *Hatschbach 56145* (US); *H.diguense* (Cuatrec.) Cuatrec., *Quizhpe et al. 612* (US); *H.glaziovii* (Urb.) Cuatrec., *Landrum 4262* (US). *Hylocarpaheterocarpa* (Ducke) Cuatrec., *Ducke [JBRJ-30137*] (US). *Sacoglottisgabonensis*, *Gentry & Pilz 32860* (US); *S.guianensis* Benth., *Carvalho et al. 4346* (US); *S.maguirei* Cuatrec., *Maguire et al. 30693* (NY, US); *S.perryi* sp. nov., *Redden 7264* (US), *Tripp 2984* (US). *Schistostemonmacrophyllus* (Benth.) Cuatrec., *Maas et al. 6577* (NY); *S.oblongifolius* (Benth.) Cuatrec., *Maas et al. 6804* (US). *Vantaneabahiensis* Cuatrec., *Belém & Magalhães 748* (US); *V.compacta* (Schnizl.) Cuatrec., *Hatschbach 21265* (NY); *V.depleta* McPherson, *Hammel & Trainer 12954* (MO), *McPherson & Stockwell 10892* (US); *V.micrantha* Ducke, *Ducke [JBRJ-30135*] (US); *V.peruviana* J.F. Macbr., *Rimachi 4577* (US); *V.spiritu-sancti* comb. nov., *Silva et al. 1436* (US). Stamen vasculature was examined after clearing with aqueous 5% (w/v) sodium hydroxide, followed by saturated chloral hydrate. For floral anatomy of *Vantaneaspiritu-sancti*, buds were cleared of tannins with Stockwell’s bleach, paraffin-embedded, sectioned at 10 μm, and stained with toluidine blue O. Stamen structure descriptions follow the work of Endress (e.g., Endress and Stumpf 1990, 1991).

Character states in Tables 1, 2 were based, except where noted, on new primary observations. Fruits vary in size and shape among *Sacoglottis* species, but distinguishing true size differences from developmental differences in their slow maturing fruits is to some degree unclear given that most fruits are collected immature. Length-to-width ratios (l/w) were used as a simple shape proxy to reflect degree of elongation in their basic ellipsoid shape, and a key character in distinguishing fruiting collections of the newly described species. Based on the many available fruits of the new species at various developmental stages (although we lack truly ripe fruit), this ratio is apparently stable across a range of sub-maturity. Similar large sample sizes were not available for most other *Sacoglottis* species. A full understanding of inflorescence structure in *Sacoglottis* with its multiple orders of often dichotomous branching is beyond the scope of our study and would be difficult to carefully assess with incomplete herbarium collections. However, we did quantify peduncle and pedicel lengths; the latter usually vary several-fold within an inflorescence.

Representative leaves from nearly all Humiriaceae species and varieties were surveyed with LM for the presence of foliar glands and leaf margin features. Type collections were utilized where possible to avoid identification uncertainty, and multiple specimens were studied, especially for taxa with glands characterized as scarce or potentially absent. In some cases, distinguishing between plant structures and leaf damage, which creates circular depressions with marginal scarring, was difficult. We employed additional search criteria of expected location and positional patterns, although the laminar glands of some species are clearly sparse and erratically distributed.

## Results

### Androecial morphology

**Figure 1.**
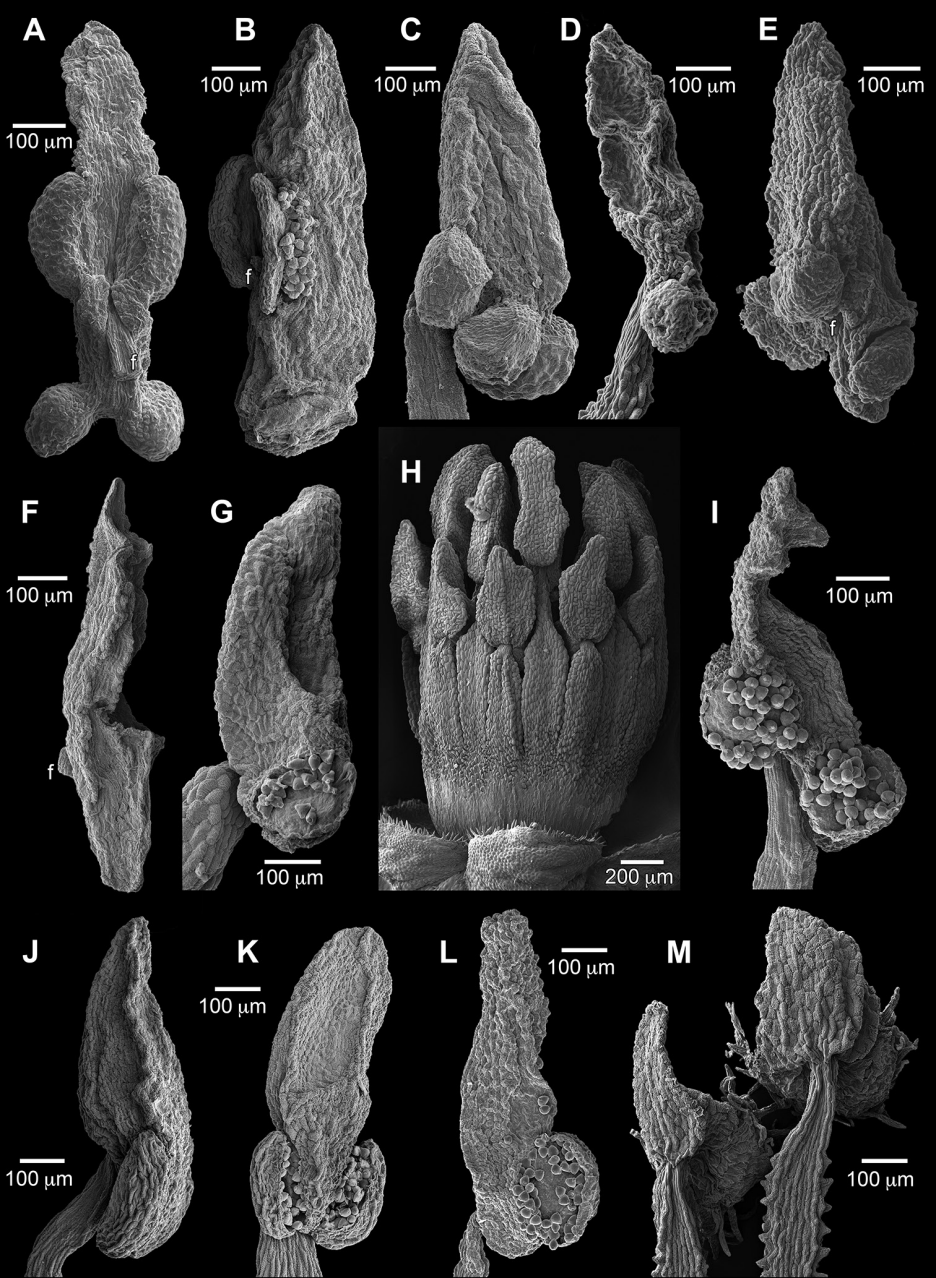
Stamen structure of Humiriaceae. **A***Duckesiaverrucosa* tetrasporangiate anther, dorsal **B***Duckesiaverrucosa* tetrasporangiate anther, lateral **C***Duckesialiesneri* tetrasporangiate anther, lateral **D***Endopleurauchi* disporangiate anther, lateral **E***Endopleurauchi* tetrasporangiate anther, lateral **F***Duckesiaverrucosa* sterile anther, lateral **G***Humiriastrumcuspidatum* disporangiate anther, lateral **H***Humiriastrumcuspidatum* androecium, dorsal **I***Endopleurauchi* tetrasporangiate anther, lateral **J***Humiriastrumdentatum* disporangiate anther, lateral **K***Humiriastrumdentatum* disporangiate anther, ventral **L***Humiriastrumdiguense* disporangiate anther, lateral **M**Humiriabalsamiferavar.imbaimadaiensis stamen cluster with 2 of 3 types, dorsal. f = filament attachment location. Sources: **A, B, F***Ducke 2108***C***Henderson 933***D, E, I***Assunção 605***G, H***Cid et al. 4264***J, K***Hatschbach 56145***L***Quizhpe et al. 612***M***Wurdack 4814* (all US.)

The flowers of *Duckesiaverrucosa* have (22–)25 stamens of two types, including five fertile antepetalous that each alternate with a group of 4–5 sterile on less stout filaments of varying lengths. One stamen per sterile group is usually as long or longer (clearly the medial antesepalous stamen when five per group) than the fertile stamens and the rest shorter. The filaments are subulate, complanate, basally very short-connate, proximally short-papillose, and distally smoother (especially so on the fertile stamens). Abnormally short filaments (1/3–1/2 of the normal length) lacking anthers rarely substitute for sterile stamens; these are likely the occasional staminodes reported by Cuatrecasas (1961). The filaments each contain a vascular bundle that continues into the connective protrusion for nearly its entire length, and is distally branched in the fertile but unbranched in the sterile anthers. Anthers are dorsifixed below midlength, glabrous, lack stomata, caducous, and have elongate connective protrusions, which are especially thickened and fleshy in the fertile anthers. The fertile anthers are tetrasporangiate, with a pair of larger sporangia (pollen sacs) positioned dorsally at the level of the filament insertion, and a pair at the base that are slightly smaller and nearly perpendicular to the dorsal pair (Fig. 1A, B). Dehiscence of the four thecae is by flap-like valves that open from the ventral side for each lateral sporangium and move dorsally (outward), or for a basal sporangium move from distal to proximal (drop downward; Fig. 1B). The sterile anthers consist of connectives with protrusions that are thinner than those of the fertile anthers and have little trace of sporangia (Fig. 1F); those connectives can be prolonged and misshapen due to packing in bud and form filling around the fertile anthers. The 20 stamens of *Duckesialiesneri* comb. nov. are of two types with five long antesepalous and 15 short. The filaments are subulate, complanate, connate for up to 1/3 of their length, smooth, and contain a vascular bundle that continues into the connective protrusion and is usually unbranched (rarely with short stubby branches distally). The anthers are all fertile, tetrasporangiate and similar to *D.verrucosa*; however, the pairs of sporangia lack size differentiation and are close together rather than well-separated (Fig. 1C). In bud, both species have stamens with straight filaments that are longer than the anthers.

The flowers of *Endopleura* have 20–22 stamens (to 30, fide Cuatrecasas 1961) of two types. Five antepetalous and five antesepalous long stamens are tetrasporangiate with separated lateral (dorsal) and basal pairs of sporangia (Fig. 1E, I). The 10+ short anthers alternating with the long type are usually disporangiate by missing the lateral pair, or rarely with no (sterile) or three sporangia (Fig. 1D). Filaments are subulate, complanate, basally very short-connate, and short-papillose nearly their entire length; each contains a vascular bundle that continues into the connective protrusion where it is unbranched or distally branched (branching does not appear correlated with stamen type). In bud the filaments are straight with slightly sinuous tips for the longer type (likely due to packing), and as long or longer than the anthers. Anthers are dorsifixed below midlength, glabrous, versatile, lack stomata, caducous, and variously shaped due to distortions of the usually thickened, elongate connective protrusions from packing in bud (e.g., Fig. 1D with impressions of adjacent sporangia indenting the connective protrusion). The four thecae and dehiscence lines resemble those of *Duckesia*.

The flowers of *Humiria* have 20 stamens of three types, including five long antesepalous, 10 short and adjacent to antesepalous, and five antepetalous of intermediate length. Two (Cuatrecasas 1961) or three (Narayana and Rao 1973b) anther types have been previously reported, and we observed three types across multiple species and varieties examined. The filaments are subulate, complanate, and connate up to half their length to form a smooth androecial tube. The free tips are smooth dorsally but otherwise papillose along their margins where the projections interdigitate, and more sparingly short-papillose ventrally. The filaments of H.balsamiferavar.imbaimadaiensis each contain a vascular bundle that continues into connective protrusion for nearly its entire length and is unbranched. Anthers are disporangiate, dorsifixed at or near their base, versatile, lack stomata, and caducous (Fig. 1M). The thickened, elongate apical protrusions are glabrous and variously shaped according to their type and packing in bud; the protrusions of the long stamens wrap over and conform to the stigmas. The two sporangia are coarsely hirsute, slightly divergent, and each dehiscent from the ventral side via a dorsal hinge (opening outward).

The flowers of *Humiriastrum* (Urb.) Cuatrec. have 20 stamens of two types, including 10 long antesepalous and antepetalous, and 10 short that are adjacent to the long (Fig. 1H). The filaments are subulate, complanate, basally short- to half-connate, and smooth to sparsely short-papillose; each contains a vascular bundle that continues into the connective protrusion for half to nearly all its length, and is unbranched (*H.diguense*) or branched (*H.dentatum*). In bud, the filaments are straight and longer than the anthers. Anthers are disporangiate, dorsifixed below midlength, delicately attached, glabrous, lack stomata, caducous, and have large, thickened apical protrusions, which for the long stamens can wrap over the stigmas with tips folded inward. Cuatrecasas (1961: 49) described the “thecae of anthers basal” in *Humiriastrum* versus “inferolateral” in *Sacoglottis* and *Schistostemon*. However, this distinction is blurred as both orientations occur in *Humiriastrum*. In *H.dentatum*, the sole species studied by Narayana and Rao (1973a), the two sporangia are slightly divergent (V-shaped), and dehiscent from the ventral side in the manner of the other disporangiate genera (Fig. 1J–K); this configuration also characterizes *H.glaziovii*. Most of the remaining species in the genus have variations on (sub)parallel (not divergent) basal sporangia. For example, the sporangia of *H.cuspidatum* (Fig. 1G) are small, positioned at the anther base so as to be perpendicular to the connective long axis, and each dehiscent by a valve opening distal to proximal (drops downward). In *H.diguense*, the sporangia of the long stamens are oriented parallel to the connective long axis, and each open from the dorsal side via a ventral hinge (Fig. 1L); the short stamens have smaller, often asymmetrically paired sporangia. This dehiscence configuration is opposite of that found in *H.dentatum*, although it also differs in other details such as sporangia not divergent.

The flowers of *Hylocarpa* (examined with LM from a single, young bud and additional fragments) have 20 stamens of two types, including 12 fertile and the rest sterile (30 stamens with 5–15 fertile according to Cuatrecasas 1961), whose exact arrangement was not determined. The filaments are straight, not obviously complanate, shorter than the anthers, and smooth; the papillose filament ornamentation reported by Cuatrecasas (1961) may occur later in development. Fertile anthers are disporangiate, dorsifixed, attached in their proximal third, glabrous, and have large, carnose, clavate connectives that are unlike the tapering apical protrusions in the other genera; sterile anthers are slightly larger with connectives that are more irregularly shaped (Fig. 3G). The basal sporangia are small and divergent on the V-shaped connective base.

**Figure 2.**
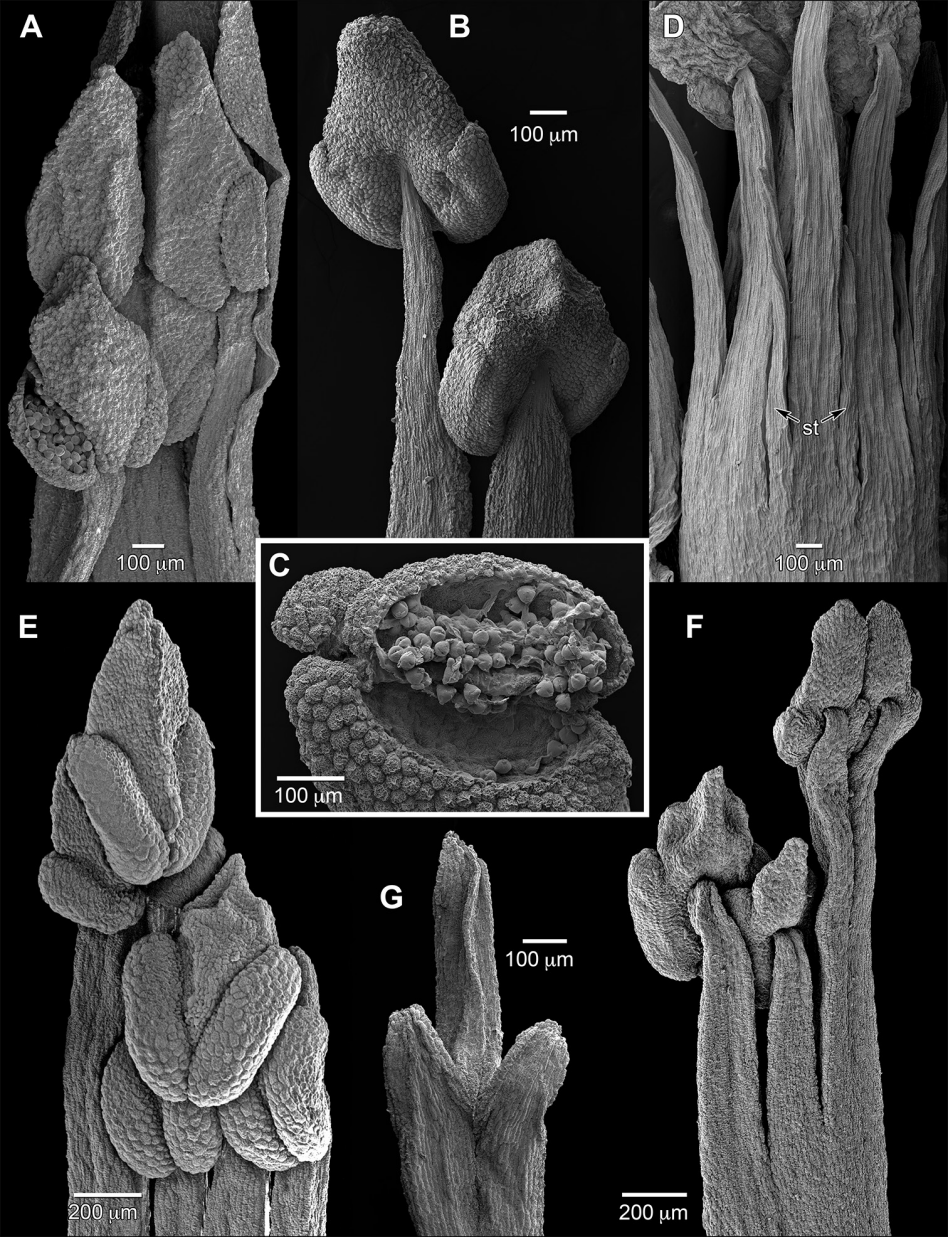
Stamen structure of Humiriaceae. **A***Sacoglottisperryi* stamen cluster of 2 types, ventral **B***Sacoglottisperryi* stamen cluster of 2 types, dorsal **C***Sacoglottisperryi* short-stamen anther with open stomium and pollen **D***Sacoglottisguianensis* androecium with interstaminal staminodes (st), dorsal **E***Schistostemonmacrophyllus* stamen cluster of 3 types, ventral **F***Schistostemonmacrophyllus* stamen cluster of 3 types, dorsal **G***Schistostemonoblongifolius* trifurcate filament tip, dorsal. Sources: **A–C***Tripp 2984***D***Carvalho et al. 4396***E, F***Maas et al. 6577***G***Maas et al. 6804* (all US).

The flowers of *Sacoglottis* have 10 stamens of two types, including five long antesepalous and five short antepetalous. The filaments are subulate, complanate, basally connate to form an androecial tube, and smooth; each contains a vascular bundle that continues into the connective protrusion for nearly its entire length and distally usually bears short stubby branches. In bud, the filaments are straight and longer than the anthers. The filament width and length of connation (1/10–2/3 of total length) vary between species and can be diagnostic (see Table 2). Anthers are disporangiate, dorsifixed near their midlength or just below, versatile via a delicate attachment, glabrous, lack stomata, and caducous (Fig. 2A, B). The two sporangia are slightly divergent (V-shaped) with dehiscence lines on the ventral side that extend over both upper and lower shoulders. The stomium of each sporangium is thus eccentric with the elliptic flap-like valve longer than its dorsal hinge which is much thinner than the thickened valve lip (Fig. 2B, C). The anther connective has a thick apical protrusion, which in bud for the short stamens is compressed against and takes on the shape of the bases of the long stamens, leading to a degree of dimorphism between the two anther-types from this space filling effect. Thin staminode-like processes were rarely observed between the stamens of *Sacoglottisguianensis* (Fig. 2D). The anthers of *S.gabonensis* are the largest in the genus (to 1.5 mm long for the long stamens; 2 mm fide Cuatrecasas 1961), but structurally otherwise resemble the other species. We reexamined the limited remaining floral material on the type of *S.maguirei* (anthers were not seen) and could confirm it has a *Sacoglottis* bauplan with 10 stamens of two alternating types; its filaments resemble those of the other species and are short, wide, and connate half their length. This species otherwise vegetatively more closely resembles small-leaved species of *Humiriastrum*.

**Figure 3.**
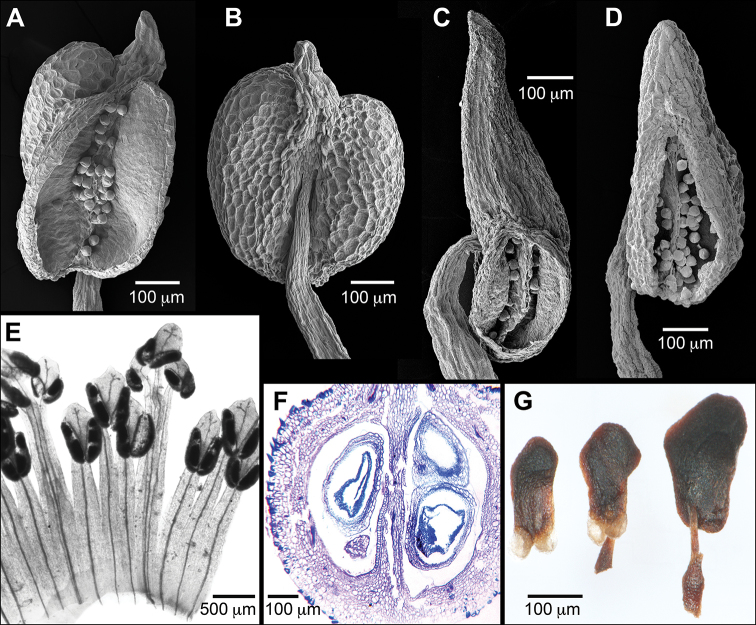
Stamen structure and anatomy of Humiriaceae. **A***Vantaneacompacta* anther, lateral **B***Vantaneacompacta* anther, dorsal **C***Vantaneadepleta* anther, lateral **D***Vantaneaspiritu-sancti* anther, lateral **E***Schistostemonoblongifolius* clearing of androecium with 3 stamen types, ventral **F***Vantaneaspiritu-sancti* longitudinal section of ovary with 2 superposed ovules per locule; lower left ovule partial **G***Hylocarpaheterocarpa* anthers (left to right): disporangiate dorsal, ventral; sterile, dorsal. Sources: **A, B***Hatschbach 21265***C***Hammel & Trainer 12954***D, F***Silva et al. 1436***E***Maas et al. 6804***G***Ducke [JBRJ-30137*] (all US).

The flowers of *Schistostemon* (Urb.) Cuatr. have 20 stamens of three types, including five long trifurcate antesepalous, 10 short and adjacent to the antesepalous, and five of medium length antepetalous, and have 30 anthers (Fig. 2E, F). The filaments resemble those of *Sacoglottis* and are subulate, complanate, basally connate to form an androecial tube, and smooth. However, the five long stamens have a trigonous (in transverse section) free portion of the filament that then distally trifurcates, with each branch bearing an anther such that each stamen has three anthers. The lateral (dorsal) branches in the plane of the staminal tube are shorter, and the central (ventral) branch is longer and bent slightly inward (Fig. 2G). The five trifurcate stamens of *S.oblongifolius* have three co-lateral vascular bundles per filament that diverge at the branched apex with one bundle serving each anther, while the remaining 15 stamens each have a single bundle; each vascular bundle continues into the connective protrusion to nearly the tip and distally usually bears short, stubby branches (Fig. 3E). The disporangiate anthers of *Schistostemon* are very similar in morphology to those of *Sacoglottis*, including in details of attachment, space-filling protrusion dimorphism, branched vasculature in the apical protrusion, slight sporangia divergence (V-shaped), and dehiscence by valves that open from the ventral side and move dorsally (outward). However, the lateral anthers of each trifurcate stamen are slightly reduced in size relative to the central one.

The flowers of *Vantanea* vary in stamen number across the species, ranging from 15–28 in *V.depleta* to >200 in *V.maculicarpa* Sabatier & J. Engel (fide Engel and Sabatier 2018), and vary in length from 3 mm in *V.spiritu-sancti* to 35 mm in *V.guianensis* Aubl. Stamen number can also vary within collections and species, and the positions of different types (lengths) relative to sepals and petals is indeterminate with higher numbers. The filaments are much longer than the anthers, thin, subcomplanate, of multiple slightly different lengths within a flower, usually distally sinuous in bud which is retained at anthesis, and can be basally connate to form discrete whorls or less ordered. In *V.peruviana*, an inner cluster is differentiated by greater connation and papillose filaments, while the outer stamens are less connate and smooth. In *V.micrantha*, stamen whorls are poorly differentiated and all filaments are smooth. In species with relatively few, short stamens (i.e., *V.depleta*, *V.spiritu-sancti*), the filaments are clearly a single file and complanate; each contains a vascular bundle that continues into the connective protrusion for half its length and is unbranched. Anthers are tetrasporangiate with two thecae, each containing an internal septum that delineates two pollen sacs per theca. They are dorsifixed at midlength to lower third, versatile, glabrous, lack stomata, and caducous; dehiscence is by a single longitudinal slit per theca that opens widely (Fig. 3A–D). Thecae are variable in size, shape, symmetry, and orientation among the species, including elongate, extending most of the length of the anther and lateral (*V.compacta*), or shorter (to subovate), limited to the lower half of the anther, and slightly divergent (V-shaped in *V.depleta*). A theca can be (mono)symmetric, or asymmetric due to size differences between the two constituent sporangia or lateral shifts of them relative to each other. The connective protrusions are thickened and elongate, with some variation according to species such that larger protrusions appear correlated with smaller sporangia (see Fig. 3A–D). In *V.spiritu-sancti*, the 20 stamens are clearly of three alternating lengths in the manner of *Schistostemon* including five long antesepalous, 10 short and adjacent to the antesepalous, and five of medium length antepetalous. Its subulate filaments are the shortest in the genus at 2–3 mm, basally connate to 1 mm, distally slightly sinuous, and minutely papillose. The anthers are similar in structure to other *Vantanea* spp., with four sporangia, internal septa, and dehiscence by longitudinal slits (Fig. 3D). In *V.depleta* we found a variable stamen number with 22–28 for *Hammel & Trainer 12954*, and verified a lower range of 15–18 for the type collection, *McPherson & Stockwell 10892*, as had been reported by McPherson (1988). Its stamen structure of alternating lengths with the longest approximately antesepalous resembles that of *V.spiritu-sancti*, although less fixed.

### Extrafloral nectaries and foliar glands in Humiriaceae

**Figure 4.**
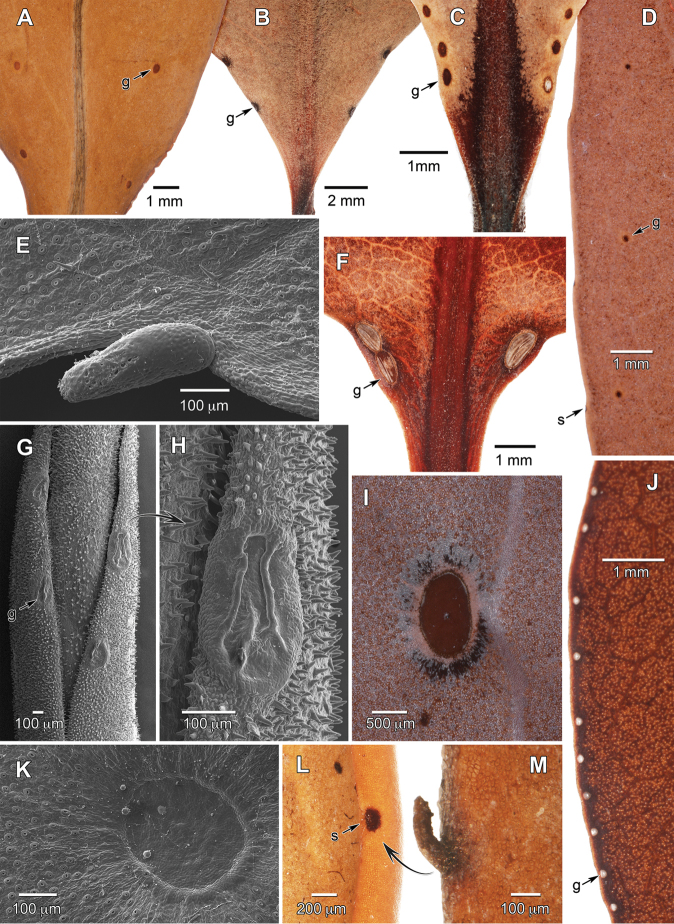
Extrafloral nectary and leaf margin diversity of Humiriaceae. **A***Vantaneadepleta* laminar glands, abaxial **B***Duckesialiesneri* basilaminar glands, adaxial **C***Schistostemonoblongifolius* basilaminar glands, adaxial **D***Humiriastrumottohuberi* laminar glands, abaxial **E***Duckesiaverrucosa* robust seta at margin **F***Sacoglottisguianensis* basilaminar glands, adaxial **G***Humiriafruticosa* shoot tip with marginal glands exposed on expanding new leaf **H***Humiriafruticosa* marginal gland **I***Hylocarpaheterocarpa* laminar gland, abaxial **J**Humiriabalsamiferavar.minarum dense row of marginal glands, abaxial **K***Duckesiaverrucosa* laminar gland, abaxial **L***Schistostemonretusus* darkened scar from deciduous seta **M***Schistostemonretusus* intact seta at margin. g = gland, s = seta scar. Sources: **A***Mori & Kallunki 4889***B***Liesner 22589***C***Maas et al. 6804***D***Maguire 34912***E, K***Ducke 2108***F***Jansen-Jacobs et al. 1898***G, H***Steyermark 103255***I***Ducke [JBRJ-30137*] **J***Mexia 5815***L***Redden 3372***M***Cuatrecasas 7203* (all US).

Foliar glands were found to be present on 64/65 accepted species and 22 infraspecific taxa of Humiriaceae (85 taxa examined, 100% presence; Appendix 1, Fig. 4). The remaining species not seen, recently described *Vantaneamaculicarpa* Sabatier & J. Engel, also has scattered abaxial glands reported (Engel and Sabatier 2018). Gland distribution ranges from relatively regular within a species, to variable between collections or even leaves on the same specimen. The two basic positional types include: (1) adaxial leaf base (basilaminar) glands as 1–3(7) more or less symmetric pairs spanning the midvein (Fig. 4B, C, F), and (2) embedded laminar glands as rows (Fig. 4A, D, J) or scattered on the abaxial (rarely also adaxial) surface (Fig. 4I). Both types of glands are circular to elliptic, sunken (especially the laminar glands) or slightly raised, have rims that are variously thickened, an outer corona free of stomata, and smooth surfaces without pores (Fig. 4H, K; 5C). In herbarium specimens the gland epidermis can be detached from the underlying gland and appear pale or whitish, which likely indicates a fluid-filled reserves pocket in the living plants (Fig. 4F, J). The extremes of gland size range from relatively large, elliptic (1.3–1.4 × 0.8–0.9 mm on *Hylocarpaheterocarpa*; Fig. 4I) to tiny, circular (<0.1 mm diameter on *Sacoglottismaguirei*) structures. The laminar glands are most frequently arranged in a row near or on the margin (Fig. 4D, J) but they can also be in a row at a lesser distance from the midvein (Fig. 4A), or more rarely widely scattered. The glands away from the margins are often clearly associated with higher order veins (i.e., secondaries, intersecondaries) or otherwise intercostal. The thickened lamina often obscure higher order venation that is likely associated with intercostal glands. The glands are very sparse and/or difficult to distinguish in *Duckesia* and some *Humiriastrum* (i.e., *H.melanocarpum* [Cuatrec] Cuatrec., *H.columbianum* [Cuatrec.] Cuatrec.), and many other taxa have only a subset of gland positions (i.e., either adaxial basilaminar or abaxial laminar). For example, the scattered abaxial laminar glands in *Duckesiaverrucosa* are only a few per specimen with most leaves lacking glands. Damage to basilaminar glands, such as appearing to be chewed, was sporadically observed and may be due to insect predation.

**Figure 5.**
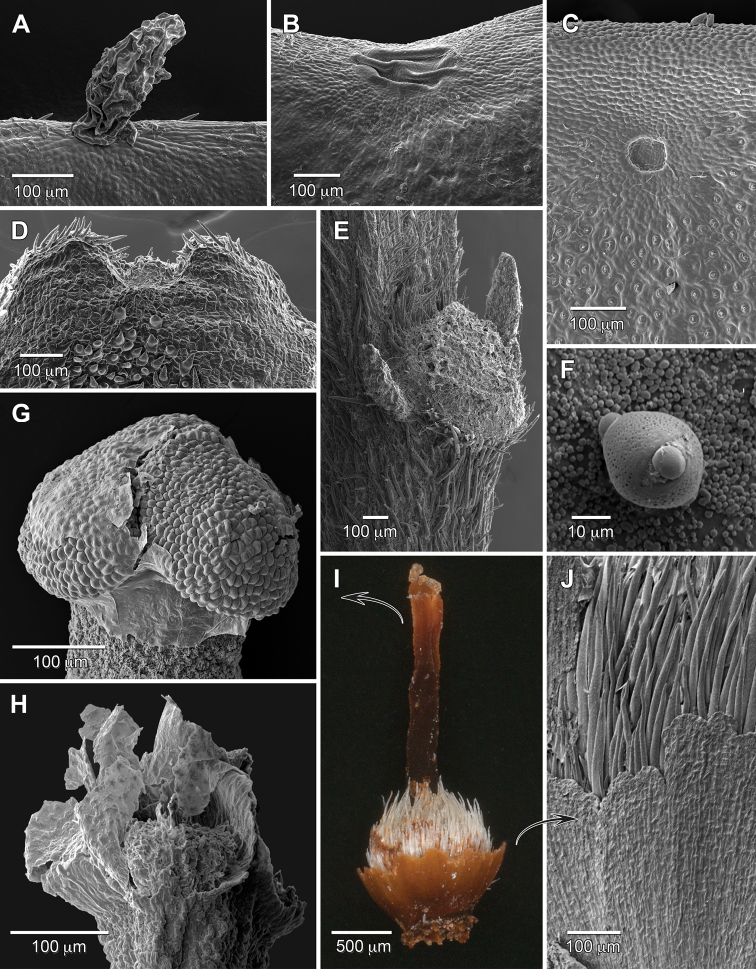
Micromorphology of *Sacoglottisperryi*. **A** Marginal glandular seta **B** basilaminar gland, adaxial **C** laminar gland near margin, abaxial **D** sepal tip, inner side with terminal gland **E** paired glandular stipules and petiole scar **F** pollen inside sporangium **G** stigma with ephemeral lobes intact and showing secretion **H** stigma lobes shredded showing thin walls **I** gynoecium with diagnostic hirsute ovary **J** glandular disc with erose margin. Sources: **A–C***Gillespie 2810***D–J***Tripp 2984* (all US).

In *Sacoglottis* the paired adaxial glands usually occur near the base of the leaf blade (Fig. 4F), and details of their position, size, and shape can differ between species. The circular basilaminar glands of *S.gabonensis*, when present (lacking on some leaves), are unusual in appearing positionally where the first proximal teeth would be along the crenate margin. However, they lack setae associated with teeth and resemble typical basilaminar glands. The other species of *Sacoglottis* have basal glands (except clearly absent in *S.maguirei*) that are more typically basilaminar and adaxial rather than marginal. Most of the other foliar glands in *Sacoglottis* are tiny, in a row near the abaxial margin, and without thickened rims (Fig. 5C), although they can be variable in size, position, and number among species. *Sacoglottisguianensis* has considerable intra- and inter-specimen variation in the number of foliar glands. The glands of *Sacoglottismaguirei* are obvious on young unexpanded leaves, and then become nearly invisible small pits on mature leaves. The deciduous stipules in *Sacoglottis* are morphologically similar to the marginal glandular setae (Fig. 5A, E).

The leaf margins are either untoothed and entire (*Humiria*, *Vantanea*), or have glandular setae at regular intervals associated with teeth of varying degrees of prominence (remaining taxa). The non-entire margins are variously described herein (Appendix 1) and in the literature as crenate(-serrate), crenulate(-serrate), or serrate; they are rarely subentire and nearly smooth with little evidence of setae. This terminology reflects a continuum among species in the height and curvature of their small teeth. Most species have asymmetric teeth that are shallow and rounded (crenate), and more rarely have pointed projections with strongly concave distal flanks (serrate). *Humiriastrummelanocarpum* with leaves described as having “the margin slightly crenate with small glands” (Cuatrecasas 1961: 154) also has typical non-entire margins but more prominent darkened attachments for the setae. The setae appear deciduous in almost all species, usually rapidly so from young expanding leaves, and leave scars with sometimes darkened, glandular-appearing points of attachment to the leaf that can be used to infer their distribution in the absence of rarely collected young leaves (Fig. 4L, M). The setae have fairly uniform morphology with modest variation in size, although those of *Duckesiaverrucosa* are distinctive in smooth cylindrical shape which is not collapsed on drying and greater persistence (Fig. 4E). In *Humiria* the abaxial glands are in a (sub)marginal row, 0–0.2 mm from the edge, and associated with slight sinuses so as to form a margin best described as repand or slightly undulate (Fig. 4J). This distinctive margin type clearly does not have setae or associated teeth. The few glands at the leaf base of *Humiriafruticosa* Cuatrec. are precociously exposed (Fig. 4G) prior to full unrolling of the young leaf (supervolute-involute ptyxis), and present an aspect not seen in other *Humiria* taxa which otherwise all have their usually more distal marginal glands hidden in tight involute laminar rolls. The 14 varieties of *Humiriabalsamifera* Aubl. span considerable variation in leaf features including in marginal gland density and lamina size.

### Taxonomic treatment

#### Duckesia liesneri

Taxon classificationPlantaeMalpighialesHumiriaceae

(Cuatrec.) K.Wurdack & C.E.Zartman comb. nov.

urn:lsid:ipni.org:names:77198710-1

Humiriastrum liesneri Cuatrec., Phytologia 71: 165. 1991. Type: VENEZUELA. Amazonas, Depto. Rio Negro: Cerro Aracamuni, summit, Proa camp, 1400 m, 28 Oct 1987, *R. Liesner & G. Carnevali 22589* (holotype: US-3111383; isotypes: MO-3641994, NY-00329209, US-3118837, VEN-277523).

##### Notes.

When Cuatrecasas (1991) originally described *Humiriastrumliesneri*, he expressed uncertainty about its anther structure and relationships due to reliance on young buds for floral details. A second collection (Venezuela. Amazonas, Sierra de Unturán, 1150 m, 3 Feb 1989, *Henderson 933*, US), correctly annotated by Cuatrecasas as *H.liesneri* in 1994, is in flower but did not cause any classification changes for the subsequent Flora of the Venezuelan Guayana (Cuatrecasas and Huber 1999). The anther morphology of this species (Fig. 1C), with its four separate sporangia, suggests an affiliation with *Duckesia* or *Endopleura*, and the transfer here to the former is supported by more similar connective morphology, absence of disporangiate anthers, and similar small leaves (Table 1). The two species of *Duckesia* are otherwise very distinct from each other; fruits remain unknown for *D.liesneri* and would likely provide additional distinguishing characters. The two collections of *D.liesneri* are ca. 60 km apart on isolated Venezuelan tepuis near the Brazilian border and north of *D.verrucosa*, which is a lowland Brazilian species.

**Table 1.** Comparison of select diagnostic features for *Duckesia and Endopleura*.

Characters	*Duckesiaverrucosa*	*Duckesialiesneri*	*Endopleurauchi*
Habit	Tree, 12–30 m (27–67 cm dbh)	Shrub to small tree, 2–4 m	Tree, (12)20–40 m (30–70 dbh)
Leaves	Petiole to 1 mm long; blade 5.5–7.5 ×1.7–2.3 cm, lanceolate, subcoriaceous; teeth setae robust, to 0.2 mm long, sometimes retained at maturity; abaxial glands sparse, 0–3 per leaf, 2/3 exmedial distance	Petiole to 2 mm long; blade 3.5–4.5 × 2.2–2.7 cm, elliptic to obovate, coriaceous; teeth setae delicate, to 0.3 mm long, rapidly deciduous; abaxial glands absent	Petiole 10–30 mm long; blade 17–28 × 5.5–6.5 cm, oblong to narrowly elliptic, subcoriaceous; teeth setae delicate, to 0.5 mm long, rapidly deciduous; abaxial glands in row near margin
Inflorescence	Axillary, small, to 1.5 cm long; bracts persistent	Axillary and subterminal, to 2.5 cm long; bracts deciduous	Axillary, to 6.5 cm long; bracts persistent
Flower	Calyx glabrous with minutely hispid margin; petals glabrous	Calyx hispid; petals coarsely hirsute	Calyx centrally coarsely hirsute, margin minutely hispid; petals coarsely hirsute
Androecium	Filaments papillose, very short-connate; anthers 5 tetrasporangiate and 15+ sterile	Filaments smooth, connate up to 1/3 length; anthers 20 tetrasporangiate	Filaments minutely papillose, very short-connate; anthers 10 tetrasporangiate and 10+ disporangiate
Gynoecium	Pistil longer than ovary	Pistil shorter than ovary	Pistil as long as ovary
Fruit	Ovoid; endocarp corrugated, 13+-radiate with densely packed woody ribs; resinous cavities small; valves conspicuous	Unknown	Ellipsoid; endocarp star-shaped, 10-radiate with 5 divided woody ribs; resinous cavities absent; valves inconspicuous
Distribution & ecology	Brazil (Amazonas especially near Manaus, Pará); 50–125 m elevation, terra firme	Venezuela (Amazonas on Cerro Aracamuni and Sierra de Unturán); 1150–1400 m elevation, upland cloud or semi-open tepui forest	Bolivia (Pando), Brazil (Amapá, Amazonas, Pará, Rhondônia) Guyana, Suriname, Venezuela; 50–620 m elevation, terra firme

**Figure 6.**
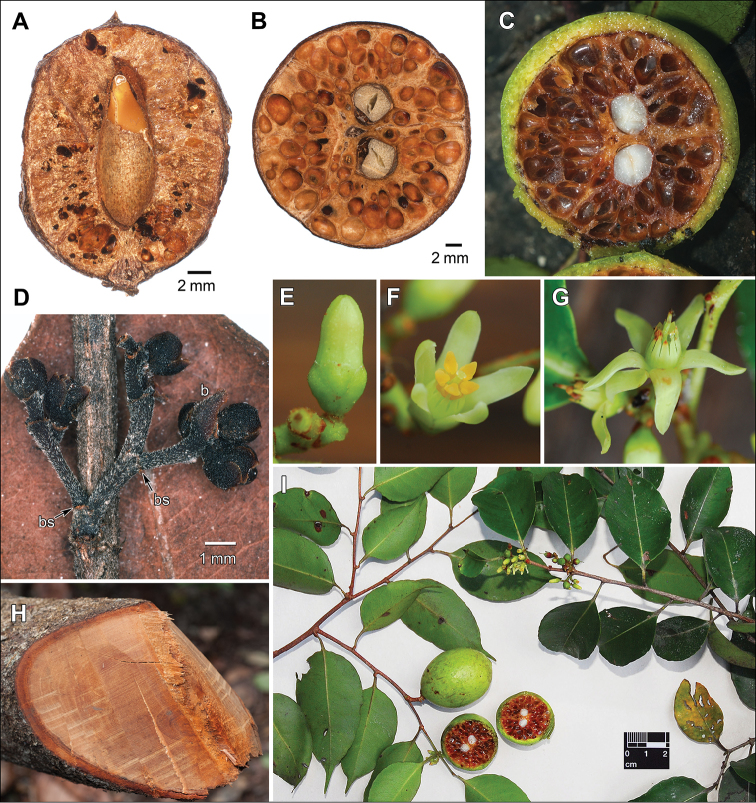
Macromorphology of *Sacoglottisperryi*. **A** Dried fruit with central seed embedded in woody endocarp, longitudinal split along carpel wall (dissected by Cuatrecasas) **B** dried fruit with 2 central seeds and endocarp lacunae, transverse section **C** fresh fruit with fleshy yellow-green exocarp and liquid in endocarp lacunae, transverse section **D** young inflorescence with bracts intact (b) or fallen leaving bract scars (bs) **E** mature bud with marginal sepal gland (small red dot in center) **F** partly open flower with intact anthers **G** post-anthetic flower **H** freshly cut trunk **I** type in life just before pressing. Sources: **A***Gillespie 2810***B***Tripp 2984***C, E–I***Redden 7264***D***Hoffman 1600* (all US).

#### Vantanea spiritu-sancti

Taxon classificationPlantaeMalpighialesHumiriaceae

(Cuatrec.) K.Wurdack & C.E.Zartman comb. nov.

urn:lsid:ipni.org:names:77198711-1

Humiriastrum spiritu-sancti Cuatrec., Ciencia, Mexico 23(4): 137. 1964. Type: BRAZIL. Espírito Santo, Mun. de Santa Tereza: Lombardia, 25 Jan 1954, *G. Dalcolmo s.n.* (holotype: RB-86212; isotype: US-2827596).

##### Notes.

*Humiriastrumspiritu-sancti* was poorly known until recent ample collections allowed a fuller characterization. Giordano and Bove (2008) described its thecae as unilocular, ovary cells as uniovulate, and fruits as having five apical foramina. The transfer here to *Vantanea* is supported by anther structure that we interpret as two lateral bisporangiate thecae with internal septa (Fig. 3D; see Results), two superposed ovules per locule (Fig. 3F), apparent lack of foramina, as well as previously reported pollen details (Bove and Melham 2000), and preliminary molecular phylogenetic placement (Zartman et al., unpublished data). Upon examination of endocarps from *Amorim et al. 1391* (US-3258470, as sectioned for Herrera et al. 2010), as well as the illustration from Giordano and Bove (2008), we could not find well-developed apical foramina that otherwise characterize *Humiriastrum*. Furthermore, the androecial bauplan of *V.spiritu-sancti* with a low number of alternating-length stamens is similar to that of *V.depleta*.

**Figure 7.**
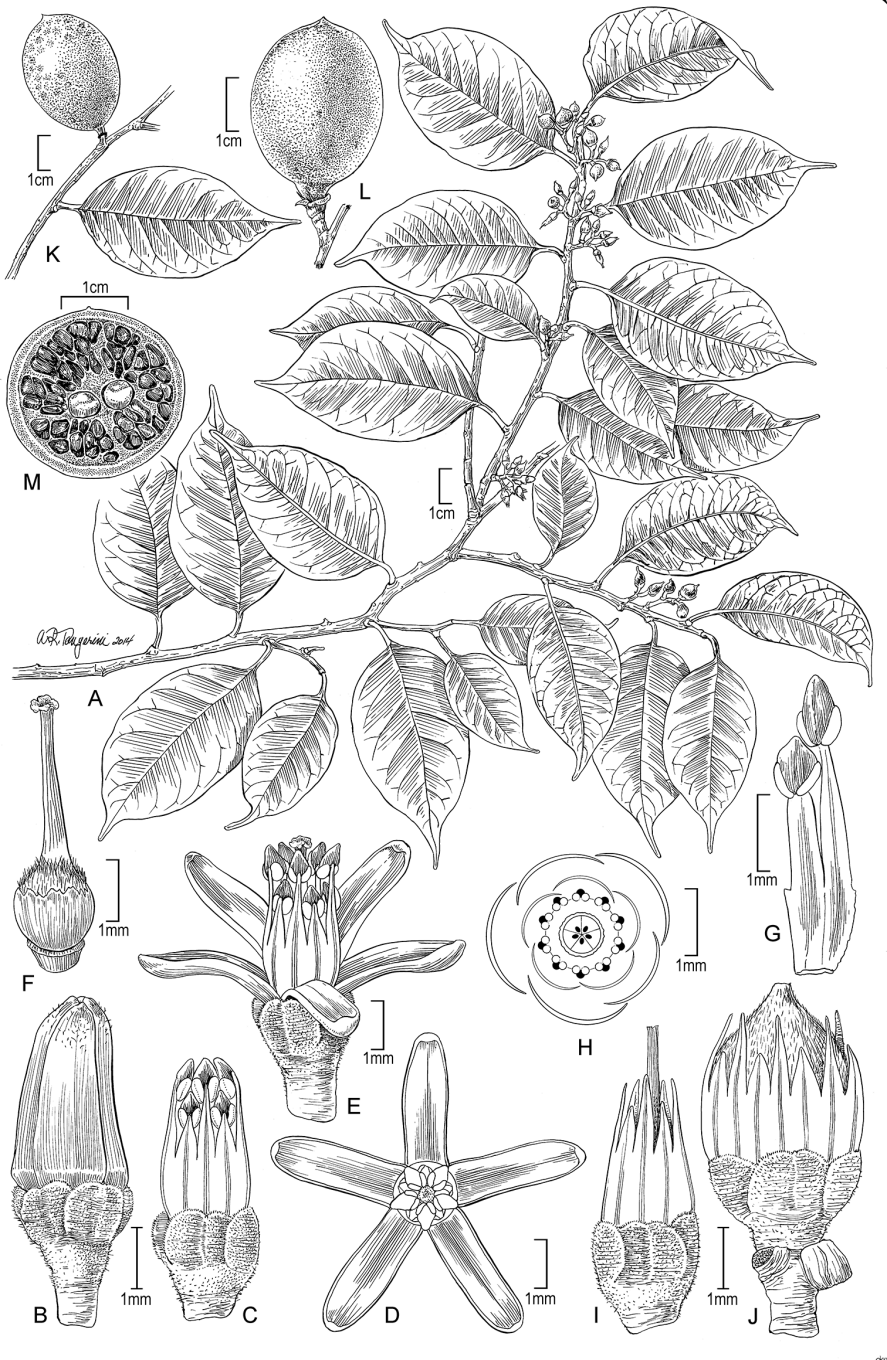
Illustration of *Sacoglottisperryi*. **A** Habit **B** bud **C** bud with petals removed **D** open flower, axial **E** open flower, lateral **F** gynoecium **G** stamen cluster of 2 types, ventral **H** floral diagram **I** post-anthetic flower **J** young fruit **K, L** fruit **M** fruit with 2 central seeds and endocarp lacunae, transverse section. Source: **A–M** from specimens and life photos of *Redden 7264* (US).

#### Sacoglottis perryi

Taxon classificationPlantaeMalpighialesHumiriaceae

K.Wurdack & C.E.Zartman sp. nov.

urn:lsid:ipni.org:names:77198606-1

Figures 5 , 6 , 7

##### Diagnosis.

Differs from *Sacoglottisguianensis* in smaller elliptic leaves, smaller short-pedunculate inflorescences with deciduous bracts, glandular sepals, hirsute ovaries, and subglobose fruits.

##### Type.

GUYANA. Cuyuni-Mazaruni Region: Below 1^st^ escarpment (of four) of Kamakusa Mt., Powis Creek (2^nd^) Camp, gallery forest, 5°48'26.7"N, 60°14'6.9"W, 662 m, 20 May 2012 [fl], *K. Redden 7264* (holotype: BRG, isotypes: K, NY, US-3694797). Note: type tree of 10 cm dbh was cut down (Fig. 6H) during sampling and provided a wood sample (K. Wurdack, personal observation).

##### Description.

*Habit* usually small tree, 6–8 m, 10–12 cm dbh (to 14 m, 50 cm dbh), trunks lacking buttresses; bark rough, scaly, inner bark coarsely fibrous, dark brown; wood reddish-brown, diffuse porous, growth rings distinct and delimited by a fibrous zone, vessels narrow, mostly solitary, tyloses present, axial parenchyma scanty paratracheal, rays conspicuous (wood described from a 4 cm diam. trunk section); lateral leafy twigs 2–3 mm diam., puberulous, trichomes to 0.1 mm long. *Stipules* ca. 0.6 × 0.3 (at base) mm, narrowly triangular (rarely with smaller secondary lobes), thickened, glandular, rapidly deciduous. *Leaves* alternate, distichous on lateral branches, simple; petioles 6–10 (long) × 1 (wide) × 0.8 (high) mm mid-petiole, subterete (dorsiventrally slightly flattened), proximally slightly pulvinate, distally expanded to 2× wider with narrow wing extending from margins of lamina along shoulders of petiole, sparsely puberulous; blades 4.4–10.8 × 1.7–4.6 cm, length:width ratio 2.19–2.59:1 (mean = 2.37, SD = 0.256, *n* = 100 from 10 mature leaves × 10 collections), oblong to ovate, base angle obtuse, base obtuse to rounded, apex angle acute, apex shape acuminate with drip tip 0.5–1.5 cm long, tip ending in minute apiculate glandular tooth at distal apex of midvein; subcoriaceous, abaxially sparsely puberulous, adaxially glabrous; basilaminar glands (0–1)2, usually symmetric as pair, along adaxial margin of leaf base, 0.2–0.5 × 0.1–0.2 mm, widely to narrowly elliptic; abaxial laminar glands sparse, up to 4 per side, 0.2–0.7 mm from edge, 0.1 mm wide, nearly circular, slightly sunken; margin shallowly crenate, darkened glandular spots in sinuses, 0.2–0.3 mm diam., when young these spots bearing deciduous glandular setae, 0.3 × 0.1 mm; dark green above and light green below in life; venation pinnate, brochidodromous; secondaries 8–9 pairs, excurrent attachment; intersecondaries frequent. *Inflorescences* axillary, small (shorter than leaves), <20 flowers, 3–4 orders of branching, peduncle to 1 mm long, rachis 5–10(20) mm long, internal internodes successively shorter in higher order branches, to 2.5 mm long on secondary branches, terminal branches (pedicels) subsessile to 0.5 mm long; bracts 1.8 × 1–1.5 mm, obtuse to rounded, margin entire, sparsely puberulous, rapidly deciduous leaving joint scars. *Flowers* bisexual, actinomorphic, mature buds 4–4.5 × 1.5–2 mm; calyx cupular, sepals 5, equal, free to base of ovary (when observed from inside), connate at base of receptacle for 1–1.5 mm, imbricate, free portion widely rounded to minutely retuse, 1 × 1.5 mm, sparsely short puberulent (trichomes shorter than elsewhere on plant) inside and out, margin finely ciliate, hyaline, usually with single gland ca. 0.1 mm diam. at minutely retuse apex, in life sepals green and gland red; petals 5, free, 4–4.5 × 1 mm, oblong-lanceolate, tip acute, thick with narrow hyaline entire margin, glabrous, aestivation quincuncial, greenish-white in life and reflexed at anthesis; stamens 10, glabrous, stiffly erect in bud and at anthesis, dimorphic, alternating in 2 lengths; 5 antesepalous longer, filaments 3.5–3.6 mm long; 5 antepetalous shorter, filaments 3 mm long; filaments of both lengths subulate, complanate, basally connate to 1.5 mm and tips free, 0.3–0.5 mm wide at start of free portion × <0.1 mm thick, in life greenish-white; anthers slightly dimorphic and differing in length of connective apex due to tight packing in bud which prevents full development of shorter anthers, antesepalous 0.8–0.9 × 0.5 mm, antepetalous 0.6–0.7 × 0.5 mm, dorsifixed; connective thickened, apical tip acute, base V-shaped and forcing pollen sacs to slightly diverge distally; pollen sacs 2 per anther (disporangiate), ca. 0.3 mm long, in proximal half of anther, stomium narrowly elliptic, covered by valve with a thin dorsal hinge and opening with a ventral lip, yellow-orange in life; ovary 1–1.2 × 1.2–1.3 mm, subglobose, densely hirsute with long trichomes to 0.5 mm; disc 0.5–0.6 mm high, thin, cupular, margin erose with rounded irregular lobes to 0.1 mm high; style 2–2.5 × 0.3 mm, single, columnar, with distinct discontinuity and slightly thinner for distal 1/3, glabrous; stigma minutely 5 lobate-capitate. *Infructescence*: 1–2 fruits maturing per inflorescence; fruit stalk 5–8 × 1 (diam.) mm, consisting of pedicel and 1–2 inflorescence nodes; petals and anthers caducous, sepals and filaments persistent, filaments forming protective palisade-like sheath around young developing fruit, stigmas and distal thinner part of style senescing rapidly. *Fruit* drupaceous, 33–35 × 23–30 mm (nearly mature, but ultimate size unknown), length:width ratio 1.07–1.44:1 (mean = 1.23, SD = 0.107, *n* = 19 from 9 collections, excluding *McDowell 2993*), sub-globose to ellipsoid, tip short apiculate, glabrescent but sparse trichomes remaining at apex, surface smooth when fresh, when dry sometimes slightly dimpled due to underlying endocarp, exocarp 0.5–1 mm thick; endocarp woody, obscurely bullate on surface due to underlying lacunae, with 3 slight longitudinal furrows, brown, interior with numerous lacunae; lacunae 1.5–5 × 1–4 mm, asymmetric ellipsoidal, greatly varying in size and exact shape within a fruit, in life filled with watery light brown fluid that dries to form thin dark brown, glassy, resinous layer; walls between lacunae <0.05 mm (translucent)–0.5 mm, grading thicker at vertices between multiple lacunae, distinctly thickened around seeds (locule cavity wall) and along the indistinct carpel sutures; locule snug around seed. *Seeds* 1–3 per fruit, 15 (long) × 5–6 (deep; parallel to embryo) × 4–5 (wide; perpendicular to embryo) mm, oblong, (sub)circular in transverse section, proximal end rounded, distal (hilar) end attenuate; coat thin, papery, brown; endosperm copious, fleshy, oily.

##### Etymology.

The specific epithet commemorates Claudius Perry (1977–2011; Kelloff et al. 2011: plate 2A), a Wapishana Amerindian originally from Marunawa, who planned to be on the type-collecting trip but tragically perished on 14 Jun 2011 when a portion of his home on the Dadanawa Ranch collapsed during a storm. Although by trade a vaquero (cowboy) in the Rupununi savanna region of southern Guyana, he also served as a parataxonomist and guide on many plant and animal research expeditions (1996–2010) across Guyana. He was especially valued in the many botanical expeditions lead by H. David Clarke (Kelloff et al. 2011), Karen Redden, and the second author. He accompanied both authors herein, had a keen eye for plant diversity, and was a highly skilled collector, especially of trees that he enjoyed rapidly climbing with spikes. He participated in expeditions that yielded the paratype collections of *Forbes 325* and *Redden 6582*, and personally gathered the latter.

##### Distribution and ecology.

*Sacoglottisperryi* is apparently confined to the Pakaraima Mountains of Guyana, and most localities are along mid-elevation (500–800 m) primary and secondary creeks in the headwaters of the Mazaruni River basin. It should be expected in other upland areas of the Mazaruni watershed including in adjacent Venezuela and perhaps Brazil. The tree typically grows in gallery forests at the edge of those waterways, or in forest patches in the white-sand savanna/forest mosaics of the region. Flowering occurs in May–June, with the fruits slowly developing over the course of a year. The timing of fruit ripening, likely in the fall, and features of the ripe fruit are unclear. The Kamakusa collections with the largest fruits on any specimens were hard and green when fresh (Fig. 6I; K. Wurdack, personal observation). In other species (e.g., *S.guianensis*; see Holanda 2013) the exocarp turns from green to deep orange, reddish, or brown, and can be fleshy and sweet. The fruits of *Sacoglottis* spp. are buoyant, leading to long distance transport by water (e.g., as ocean drift); they have also been documented to be dispersed by bats (Lobova et al. 2009) and monkeys (van Roosmalen 1985). The accessible position of the fruit of *S.perryi* on thin, pendulous, distichous-leaved branchlets over small waterway corridors appears advantageous for both bat and water dispersal.

##### Additional collections examined.

**GUYANA. Cuyuni-Mazaruni Region**: Imbaimadai, Partang River along riverbank past first rapids NE of base camp, 05°42'10.5"N, 60°16'50.1"W, 873 m, 2 Dec 2002 [fr], *Forbes 325* (US); Maipuri Falls, Karowrieng River, 05°41'N, 60°14'W, 570–600 m, 20 Dec 1989 [fr], *Gillespie 2810* (US); Imbaimadai Creek, 1 km W of Imbaimadai, 05°42'N, 60°18'W, 500 m, 16 May 1992 [fl, fr], *Hoffman 1600* (NY, US); basecamp 8.6 km NE [of] Imbaimadai on Partang River tributary, 0.75 km E, 05°46'N, 60°15'W, 625 m, 20 May 1992 [fl, fr], *Hoffman 1745* (MO, NY, US); basecamp 8.6 km NE Imbaimadai on Partang River tributary, 1.25 km E, 05°46'36"N, 60°15'49"W, 600 m, 20 May 1992 [fl], *Hoffman 1755* (MO, NY, US); Mt. Aymatoi (sandstone), 05°55'N, 61°W, 1150 m, 16 Oct 1981 [fr], *Maas et al. 5753* (MO, US); Imbaimadai Savannas, Upper Mazaruni River, 24 Oct 1951 [fr], *Maguire & Fanshawe 32250* (MO, NY); Sagaraimadai Savanna, Upper Mazaruni River, 16 Nov. 1951 [fr], *Maguire & Fanshawe 32619* (MO, NY); from Utshe River to Great Falls on Kamarang River, 4–5 km SE of Utshe camp, 05°43'N, 61°07'W, 850–975 m, 26 May 1990 [fl], *McDowell 2920* (MO, NY, US); 7 km N of Paruima Village, after descent from south face of Mt. Waleliwatipu, 05°54'N, 61°02'W, 980–1060 m, 30 May 1990 [fl, fr], *McDowell 2993* (MO, NY, US-2 sheets); to plateau [at] S end of Haiamatipu, 05°28'N, 60°32'W, 610–914 m, 20 Jun 1991 [fl], *McDowell 4734* (NY, US); Imbaimadai Creek, W of Imbaimadai, 05°42'N, 60°18'W, 503 m, 22 Jun 1986 [fr], *Pipoly 7990* (MO, NY, US); Vicinity of Chinoweing Village, 5°32'N, 60°07'W, 650–670 m, 21 Feb 1987 [fr], *Pipoly 10484* (NY-2 sheets, US); Imbaimadai, Karowrieng River, towards waterfall 2.32 mi E of Base Camp 1, bordering Karowrieng Creek, 5°40'42.4"N, 60°14'30.8"W, 575 m, 22 Jan 2004 [fr], *Redden 1489* (US); Mazaruni River, just above ABC Falls, trail/track 2.03 mi SW of Base Camp 6 heading E, 6°4'25.2"N, 60°39'14.3"W, 605 m, 19 Feb 2004 [young fr], *Redden 2008* (US); Kako River, top of waterfall, 05°28'50.8"N, 60°50'49.3"W, 687 m, 15 May 2009 [fl, fr], *Redden 6582* (US); below 1^st^ escarpment (of four) of Kamakusa Mt., Powis (2^nd^) Camp and vicinity, along creek bank at camp 5°48'34.6"N, 60°14'21.5"W, 651 m, 21 May 2012 [fl, fr], *Tripp 2984* (US); Kako River, gallery forest near rapids, 05°30'27"N, 60°50'30.3"W, 505 m, 10 May 2009 [fl], *Wurdack 4911* (US); below 1^st^ escarpment (of four) of Kamakusa Mt., Powis (2^nd^) Camp and vicinity, along creek bank at camp 5°48'34.6"N, 60°14'21.5"W, 651 m, 13 Jun 2012 [young fr], *Wurdack 5898* (US).

## Discussion

### *Sacoglottis*, and delimitation and morphology of newly described *S.perryi*

The 11 species of *Sacoglottis* are compared in Table 2 (gland details in Appendix 1), which highlights important diagnostic characters such as inflorescence structure (peduncle and bracts), sepal glands, ovary vestiture, and fruit shape. Especially significant is the densely hirsute ovary, a feature that only occurs in *S.perryi* (Fig. 5I) and Central American *S.trichogyna* Cuatrec. All other species in the genus have glabrous ovaries. Besides ovary vestiture, no other aspect of morphology suggests a closer relationship between *S.perryi* and *S.trichogyna*, and their fruit structures are very different (Herrera et al. 2010: fig. 2N). The leaves of *S.perryi* are the second smallest in the genus after diminutive *S.maguirei*, and have a very pronounced drip tip and an shallowly crenate margin (Fig. 6I, 7A). *Sacoglottisperryi* possesses the full suite of glandular structures known for the family, including small abaxial and basilaminar glands, rapidly deciduous marginal setae, thickened stipules similar to the setae, gland-tipped sepals, a floral disc, and thickened connectives (Fig. 5A–E, J). The minute stigmas appear to secrete and consist of five hollow (presumably fluid-filled in life) thin-walled lobes that rapidly degrade atop a sturdy, persistent style (Fig. 5G, H). This unusual morphology appears typical for the genus and the family in general. The small, subspheroidal, tricolporate, microreticulate pollen of *S.perryi* (Fig. 5F) resembles that of other species in the genus (see Bove and Melhem 2000). Four species of *Sacoglottis* are now known from Guyana including *S.amazonica* Mart., *S.cydonioides* Cuatrec., *S.guianensis*, and *S.perryi*. The fruit shape of *S.perryi* is subglobose and intermediate between globose *S.cydonioides* and more elongate *S.amazonica* and *S.guianensis*. The fruits of *McDowell 2993* are more elongate (length:width ratio 1.37–1.63:1, mean = 1.47, SD = 0.108, *n* = 4) than those typical for *S.perryi*, but in other characters the collection agrees with the species. The endocarp morphology of *S.perryi* does not resemble that of a putatively undescribed *Sacoglottis* only known from well-weathered ocean drift endocarps (van der Ham et al. 2015).

The biogeography of *Sacoglottis* includes disjunctions with clear long-distance dispersal to Africa for *S.gabonensis* and to Isla del Coco far off the Pacific coast of Costa Rica for endemic *S.holdridgei* Cuatrec., and there is direct evidence of contemporary and fossil sea drift (Herrera et al. 2012, 2014; van der Ham et al. 2015). Mostly Amazonian *Sacoglottisamazonica* occurs in Delta Amacuro, Venezuela and reaches the nearby island of Trinidad. Otherwise the genus and indeed the entire family oddly lack a West Indian presence (Acevedo-Rodríguez and Strong 2012). *Sacoglottisperryi* is geographically relatively isolated from other species in the genus. It only overlaps with *S.guianensis* based on a single collection (*Gillespie 2939*, US) from the vicinity of Imbaimadai that has typical elongate fruit and a long-pedunculate inflorescence. Otherwise, *S.guianensis* occurs further south in the Essequibo River watershed (i.e., southern Pakaraima Mountains and Acari Mountains on the border with Brazil) and is broadly distributed elsewhere in northern South America. We reexamined the identity of the Schomburgk brothers’ collections of *S.guianensis* ([*Rob.] Schomburgk, 2^nd^ ser. 571* but likely mistranscribed for *574*, P; *574*, F, G, NY, P; *Rich. Schomburgk 842*, F image ex B, US) from Guyana, as they collected in or near the Pakaraima Mountains, and the lectotype, *Schomburgk 574* (Cuatrecasas 1961), is from “Roraima” at the drainage divide of the Mazaruni watershed. All of these specimens have long-pedunculate inflorescences and cannot be confused with *S.perryi*. The Schomburgk expedition routes, including the approach to Mt. Roraima, skirt around the Mazaruni headwaters and were not within the presently known range of *S.perryi* (Roth 1923, van Dam 2002). A sterile collection (*Guppy 308*, NY) from southern Guyana, attributed by Cuatrecasas (1961) to *S.amazonica*, appears based on leaf similarities to be *S.guianensis*. The Humiriaceae of Guyana now totals six genera and 13 species (Funk et al. 2007).

The significance of inflorescence structure variation in *Sacoglottis* has not been previously emphasized, although it is implicit in details of the descriptions and key of Cuatrecasas (1961). His first species-key couplet relates to deciduous versus persistent bract condition, which appears correlated with other aspects of inflorescence architecture. Taxa with persistent bracts have long-peduncled, often large inflorescences, while those with deciduous bracts have short to non-existent (giving the appearance of multiple inflorescences per leaf axil) peduncles and generally smaller inflorescences. The deciduous bracts are rapidly lost, and bract morphology can be hard to document without very young inflorescences. An exception to this inflorescence structure dichotomy is *Sacoglottisgabonensis*, which has deciduous bracts and a long pedunculate (1–3 cm) inflorescence. *Sacoglottismaguirei*, a tepui endemic known only from the type collection (*Maguire et al. 30693*), has been mischaracterized as deciduous (Cuatrecasas 1961), but possesses previously overlooked very short, persistent bracts that are consistent with its long-peduncled inflorescence architecture. The bract condition for the other genera of Humiriaceae is persistent (*Endopleura*, *Humiria*, *Hylocarpa*), deciduous (*Vantanea*), or mixed (*Duckesia*, *Humiriastrum*, *Schistostemon*).

*Schistostemon*, presently containing nine species, was formerly a subgenus or section of *Sacoglottis* and elevated to a genus principally based on differences in androecial structure with a doubling of stamen number to 20, an increase to 30 anthers, and three stamen types instead of two (Cuatrecasas 1961). Of particular note in differentiating *Schistostemon* are the antesepalous longest stamens that are trifurcate at their apex to support three anthers per stamen (Fig. 2G, 3E). An overlooked difference between the genera is in pistil morphology, which is thin and as long or longer than the stamens in *Sacoglottis*, but in *Schistostemon* is stout and shorter than the stamens. Fruit and vegetative details are otherwise strikingly similar between the genera. The renewed suggestion by Herrera et al. (2010) of returning to a broader, pre-Cuatrecasas circumscription of the *Sacoglottis-Schistostemon* group (i.e., *Sacoglottis* s.l.) needs further study within the framework of a well-sampled molecular phylogenetic analysis that includes taxa with both types of inflorescence structure. Limited molecular phylogenetic evidence has indicated that *Schistostemon* is not monophyletic and embedded within a paraphyletic *Sacoglottis* (Bardon 2015).

**Table 2.** Comparison of select diagnostic features among all 11 species of *Sacoglottis*.

Characters	*amazonica*	*ceratocarpa*	*cydonioides*	*gabonensis*	*guianensis*	*holdridgei*	*maguirei*	*mattogrossensis*	*ovicarpa*	*perryi*	*trichogyna*
Inflorescence structure, peduncle/pedicel lengths	Short pedunculate, 5–6 / 0.3–1 mm	Very short pedunculate, <1 / < 0.5 mm	Long pedunculate, 20–40 / 0.5–2 mm	Long pedunculate, 10–30 / <0.5 mm	Pedunculate, (2)5–35 / 0.3–3 mm	Short pedunculate, few flowered, 1–4 / 0.2–1 mm	Pedunculate, 7–15 / 1–4 mm	Long pedunculate, 15–40 / 1.5–4.5 mm	Short pedunculate, 1–2 / < 0.5 mm	Very short pedunculate, to < 1 / < 0.5 mm	Pedunculate, clustered, 3(–6) / 0.1–0.5 mm
Bracts	Deciduous; triangular, carinate	Deciduous; shape unseen	Persistent; acute	Deciduous; triangular, carinate	Persistent; acute	Deciduous; rounded	Persistent; short, rounded	Persistent; acute	Deciduous; acute, carinate	Deciduous; obtuse to rounded	Deciduous; rounded
Sepal features	Multiple tooth-like marginal glands per sepal; margin short ciliate	No glands; sparsely short puberulent	No glands; glabrous, margin ciliate	No glands; sparsely hirsute, margin ciliate	No glands; glabrous, margin ciliate	No glands; glabrous, margin short ciliate	No glands; hispidulous	No glands; glabrous	0–1 apical gland per sepal; sparsely short puberulent, margin short ciliate	1 apical gland per sepal; sparsely short puberulent, margin ciliate	No glands; glabrous to sparsely pubescent
Petal vestiture	Glabrous	Glabrous	Hispidulous, fide Cuatrec.	Hirsute (subsericeous, fide Cuatrec)	Glabrous to puberulous	Glabrous	Hispidulous.	Glabrous	Glabrous	Glabrous	Glabrous
Filament connation (relative length)	Connate 1/3	Long connate 2/3	Connate 1/2	Short connate <1/5 (barely beyond sepals)	Connate 1/3	Connate 1/2	Connate 1/2	Connate 1/4	Connate 1/2	Connate 1/3	Very short connate ca. 1/10
Ovary vestiture	Glabrous	Glabrous	Glabrous	Glabrous	Glabrous	Glabrous	Glabrous	Glabrous	Glabrous	Hirsute	Hirsute
Fruit shape; average length:width ratio	Ellipsoid; 1.4:1	Subfusiform, elongate; ca. 2.35:1	Globose; 1.02:1	Subglobose; 1.17:1	Ellipsoid-oblong; 1.6:1	Ellipsoid; 1.3:1	Subglobose; ca. 1.1:1 (very young fruit only)	Globose; 0.92:1	Ellipsoid; ca. 1.2:1 (partly dissected fruit)	Subglobose to ellipsoid; 1.23:1	Ellipsoid, large; 1.43:1
Exocarp thickness	1.5 mm	1 mm	1–2 mm	1 mm	1–2 mm	1–2.5 mm	Unknown	(0.8)1.5–2 mm	Thick, to 5 mm	0.5–1 mm	Thick, 3–6 mm
Distribution	Brazil (Amazonas, Pará), Venezuela (Delta Amacuro), Trinidad	Columbia (Vaupes), Brazil (Amazonas), Venezuela (Amazonas)	Brazil, Guyana, Surinam, Venezuela	Sierra Leone to Angola	Brazil, French Guiana, Guyana Surinam, Venezuela,	Costa Rica (Isla del Coco)	Venezuela (Amazonas: Cerro Yapacana)	Brazil (Bahia, Pará)	Colombia (Valle del Cauca)	Guyana (Cuyuni-Mazaruni: Pakaraima Mtns.)	Nicaragua, Costa Rica, Panama

### Broader significance of androecial structure in Humiriaceae

Humiriaceae share stamens partly connate, filaments subulate and complanate, and anthers that are dorsifixed, versatile, caducous, lacking stomata, and possessing connective protrusions. Most of these shared androecial features are broadly distributed, even in combination, across the rosids (Endress and Stumpf 1991). The eight genera presently can be clearly defined based on details of androecial structure including number and morphology of both stamens and sporangia, although ovule number and endocarp structure are also important distinguishing generic characters. Our observations on gross androecial structure largely agree with and complement prior floral anatomy studies by Narayana and Rao (1969–1977). Variations in anther sporangium morphology are especially interesting from an evolutionary perspective in Humiriaceae due to transitions among the three distinct forms which include, (1) tetrasporangiate-dithecal with two lateral disporangiate thecae, a typical rosid anther configuration, in *Vantanea*, (2) tetrasporangiate-tetrathecal with four monosporangiate thecae arranged as two superposed pairs in *Duckesia* and *Endopleura*, and (3) disporangiate-dithecal with two monosporangiate thecae arranged as one pair in the remaining five genera. Disporangiate anthers characterize some families of rosids (e.g., Cucurbitaceae, Malvaceae), but are otherwise very sparse in Malpighiales (e.g., cleistogamous flowers of *Viola*, Violaceae; Endress and Stumpf 1991) except for Humiriaceae.

*Vantanea* has long been considered the most ancestral genus of Humiriaceae based on androecial structure (Cuatrecasas 1961, Narayana and Rao 1969, 1977, Herrera 2010, Kubitzki 2014), although molecular phylogenetic evidence suggests a nested placement (Bardon 2015). A complex interpretation by Narayana and Rao (1976a, b, 1977) on the origin of the disporangiate anthers based on the position of the sporangia relative to the enlarged connective in bud transverse sections posits that in *Humiria* and *Schistostemon* the dorsal (outer) sporangia were lost, while in *Humiriastrum* and *Sacoglottis* the ventral (inner) sporangia were lost. We observed the anthers of *Sacoglottis* and *Schistostemon* to be nearly indistinguishable when finely comparing anthers of the same type and did not find any transverse shifts in sporangial position to suggest differing origins. Moreover, anther similarities in regard to sporangial position and dehiscence lines extend across all the disporangiate genera (in part for *Humiriastrum*), although differences exist in connective details. *Humiriastrum* is now more homogeneous with our generic transfers (i.e., *Duckesialiesneri* and *Vantaneaspiritu-sancti*); however, the remaining species have two different thecal orientations, which could correspond to different origins (i.e., from dorsal or ventral sporangia). While *H.dentatum* and *H.glaziovii* in particular differ from the rest in regard to orientation, by other morphological bases they firmly belong to *Humiriastrum* and it is premature to reconsider generic affiliation. Whether the disporangiate bauplan has one or more origins needs further testing with floral development and phylogenetic studies. The superposed pairs of sporangia (tetrasporangiate-tetrathecal) in *Duckesia* and *Endopleura* have been thought to represent an intermediate state in the transition from tetrasporangiate-dithecal to disporangiate-dithecal anthers (Cuatrecasas 1961). Moreover, they establish homologies and clearly show that the ventral sporangia can assume a basal position with distal-proximal dehiscence. Some phylogenetic evidence has indicated those genera may be embedded within a disporangiate clade and may not be sister groups (see Herrera et al. 2010, Bardon 2015), although we found no major morphological differences to contradict a single origin of the superposed-sporangia anther type. The disporangiate and superposed-sporangia anthers of Humiriaceae, sometimes described as “dehiscing by detachment” (Cuatrecasas 1961, Kubitzki 2014), have markedly eccentric stomia that fit modern definitions of valvate and have similarities in dehiscence lines to established valvate taxa such as *Hamamelis* L. and *Grubbia* P.J. Bergius (Hufford and Endress 1989, Endress and Stumpf 1990). Their fundamentally valvate nature has not been noted in prior broad comparative anther surveys and apparently does not occur in any other rosid (see Endress and Stumpf 1990, 1991).

Disporangiate-dithecal anthers are often correlated with specialized floral biology, which can be enabled by restrictive anther openings such as valves (Endress and Stumpf 1990). The pollination biology of Humiriaceae has been only carefully studied for *Humiriabalsamifera* (Holanda et al. 2015), and indicated bee pollination of its nectariferous flowers. For the valvate taxa (i.e., all except *Vantanea*), further study is needed to see if the anther valves constrain pollen release. The greatest floral variation (i.e., perianth size, stamen number and length, color) occurs in *Vantanea* with its relatively unspecialized anthers. *Vantaneaguianensis* in particular with its showy red flowers in large clusters is likely hummingbird pollinated and recorded as visited by hummingbirds by *Gentry & Stein 46932* (US). However, it differs from typical flowers with that pollination syndrome in not being especially tubular in nature with spreading 30–40 mm long petals, which are shed early before the androecium, and stamen connation for only 5–7 mm of their 25–35 mm length. Variable morphological features that likely affect pollination biology and need further investigation in an evolutionary framework include flower color (white to greenish or red to pink), disc morphology (cupular to variously dissected), filament ornamentation (papillose to smooth), stamen length (long and thin to short and robust), stamen number (10–200+), staminodes (presence or absence), stamen connation (nearly free to half connate), anther ornamentation (hairs or connective elaborations), and mode of dehiscence (longitudinal slits or valvate). Most taxa have elaborated and likely glandular connectives and discs. Stamen connation can affect pollinator nectar access by forming a tube around the disc that varies in height among genera and species (e.g., *Sacoglottis* spp., Table 2), although nectar robbing by piercing the tube is known for *Humiria* (Holanda et al. 2015). Anthesis is likely generally of short duration based on the morphology patterns of versatile, caducous anthers and delicate stigmas, although this has been directly little studied (Holanda et al. 2015).

### Extrafloral nectaries of Humiriaceae

We documented EFNs in all species of Humiriaceae, and found distributional and morphological differences. Some of these differences appear informative at the generic level, including the marginal glands of *Humiria*, entire eglandular margins of *Vantanea*, and the exceptionally large laminar glands of *Hylocarpa*. Our EFN survey, although mostly qualitative in nature, also reveals that their species-level taxonomic value is limited due to high variation in abundance and position, and low intra-specific morphological variation. The EFN ubiquity indicates that they are phylogenetically conserved (i.e., plesiomorphic) for the family, and is a conclusion that differs greatly from character reconstructions that could be hypothesized under the prior understanding of their taxonomic distribution in only three species. This conclusion is also important when considering EFN evolution in the broader context of Malpighiales where they have been documented in 13/39 families (Weber and Keeler 2013), and when considering correlations between EFNs and higher lineage diversification rates (Marazzi and Sanderson 2010, Weber and Agrawal 2014). Humiriaceae contains relatively low extant species-richness despite EFN richness. The gross morphology and variation of Humiriaceae EFNs are consistent with reports from other families (e.g., Tilney and van Wyk 2004, Marazzi et al. 2012, Gonçalves-Souza et al. 2016), although the glands appear unremarkable with no specialized pores or unusual locations on the plants. The shallow teeth forming the crenations along the leaf margins of *Sacoglottisgabonensis* have been studied in detail, and on the young leaves were found to secrete nectar that attracts ants (Belin-Depoux 1993). Belin-Depoux (1993) did not comment on the basilaminar glands, which we found to be relatively easy to overlook and confuse with the marginal setae scars (see Results). The glands along the leaf margins of *Humiria* differ from those elsewhere in the family in their position as an often dense submarginal row. They are not modified teeth or setae, which are lacking, but appear to be typical laminar glands developmentally shifted to the margin. *Humiria* spp. are largely savanna dwellers unlike most of the other Humiriaceae taxa in forests, and habitat shifts may have influenced their EFN evolution. Such habitat shifts have been implicated in leaf defense adaptive evolution, including decrease in EFN abundance and increase in glandular trichomes in neotropical Bignoniaceae (Nogueira et al. 2012). In addition to the extrinsic defenses conferred with the glands, Humiriaceae leaves appear rich in intrinsic defenses due to their thickened and tannin-rich nature.

## Supplementary Material

XML Treatment for Duckesia liesneri

XML Treatment for Vantanea spiritu-sancti

XML Treatment for Sacoglottis perryi

## Appendix 1

**Table:** Qualitative survey of Humiriaceae for extrafloral nectaries and leaf margin features. For abaxial glands not near margins, relative exmedial distances (midvein to margin) are indicated where patterns are apparent.

Taxon	Abaxial glands	Basilaminar (adaxial) glands	Leaf margin features	Representative voucher(s)
***Duckesia***				
*D.liesneri* (Cuatrec.) K. Wurdack & C.E. Zartman	Absent	1–2 pairs at margin	Crenate, setae present	*Liesner & Carnevali 22589* (US, holotype)
*D.verrucosa* (Ducke) Cuatrec.	Sparse, 0–3 per leaf, 2/3 exmedial distance	Absent	Crenate, setae present, large	*Ducke 2106* (US)
***Endopleura***				
*E.uchi* (Huber) Cuatrec.	Row near margin	Absent	Crenate, setae long and scars large	*Assunção 605* (US)
***Humiria***				
H.balsamiferavar.coriacea Cuatrec.	Marginal, along proximal third	Absent	Entire	*Cardona 1823* (US, holotype)
H.balsamiferavar.floribunda (Mart.) Cuatrec.	Marginal, along entire length	Absent	Entire	*Maguire & Politi 27974* (US)
H.balsamiferavar.guaiquinimana Cuatrec.	Marginal, along proximal third	Absent	Entire	*Cardona 1112* (US, holotype)
H.balsamiferavar.guianensis (Benth.) Cuatrec.	Marginal, along entire length	Absent	Entire	*Schomburgk 270* (US, isotype)
H.balsamiferavar.iluana Cuatrec.	Marginal, along proximal half	Absent	Entire	*Maguire 33388* (US, holotype)
H.balsamiferavar.imbaimadaiensis Cuatrec.	Marginal, along proximal half	Absent	Entire	*Maguire & Fanshawe 32158* (US, holotype)
H.balsamiferavar.laurina (Urb.) Cuatrec.	Marginal, along entire length	Absent	Entire	*Schomburgk 560* (US, isotype)
H.balsamiferavar.minarum Cuatrec.	Marginal, along entire length	Absent	Entire	*Mexia 5815* (US, holotype)
H.balsamiferavar.parvifolia (A. Juss.) Cuatrec.	Marginal, along proximal half	Absent	Entire	*St. Hilaire* (US, isotype fragment)
H.balsamiferavar.pilosa (Steyerm.) Cuatrec.	Marginal, along entire length	Absent	Entire	*Steyermark 60289* (NY, isotype)
H.balsamiferavar.savannarum (Gleason) Cuatrec.	Marginal, along entire length or proximal half	Absent	Entire	*Tate 330* (NY, type); *J. Wurdack & Monachino 41380* (US)
H.balsamiferavar.stenocarpa Cuatrec.	Marginal, along entire length	Absent	Entire	*Maguire & Maguire 40105* (US, holotype)
H.balsamiferavar.subsessilis (Urb.) Cuatrec.	Marginal, dense along entire length	Absent	Entire	*Spruce 2454* (US, isotype)
*H.crassifolia* Mart.	Marginal, sparse, denser at base and apex	Absent	Entire	*Huber & Fernandez 11331* (US)
*H.fruticosa* Cuatrec.	Marginal, 2–4 per side near base	Absent	Entire	*Maguire et al. 36580* (US, holotype)
*H.wurdackii* Cuatrec.	Marginal, along entire length, widely spaced; absent on very narrow forms	Absent	Entire	*J. Wurdack & Adderley 42760* (US, holotype); *Huber 4842* (US), narrow-leaved
***Humiriastrum***				
*H.columbianum* (Cuatrec.) Cuatrec.	Sparse abaxial; absent on type, save for a few erratic structures that could be damage.	Absent	Serrate, setae long	*Lamb 141* (US, holotype); best gland evidence is on *Gentry & Stein* 46898 (US); *Romero Castañeda 4942* (US)
*H.cuspidatum* (Benth.) Cuatrec.	Small, sparse, near margin	1 pair, elliptic	Crenate, setae long and thin	*J. Wurdack & Monachino 40881* (US)
H.cuspidatumvar.glabriflorum (Ducke) Cuatrec.	Small, sparse, near margin	1 pair at margin (sometimes a single gland)	Serrate, setae scars	*Ducke [JBRJ-23436*] (US, isotype)
H.cuspidatumvar.subhirtellum Cuatrec.	Small, sparse, near margin	0–1 pair	Serrate, setae present	*Fróes 25463* (US)
*H.dentatum* (Casar.) Cuatrec.	Small, sparse, near margin	1 pair at margin, nearly abaxial	Serrate, setae present	*Hatschbach 4294* (US)
*H.diguense* (Cuatrec.) Cuatrec.	Along secondary vein loops toward margin	1–3 pairs at margin, nearly abaxial	Crenate, setae scars	*Cuatrecasas 14956* (US, isotype)
H.diguensevar.anchicayanum (Cuatrec.) Cuatrec.	Along secondary vein loops toward margin	1 pair at margin, nearly abaxial	Crenate, setae scars	*Cuatrecasas 14418* (US, isotype)
H.diguensesubsp.costaricense Cuatrec.	Along secondary vein loops toward margin, relatively sparse	1 pair	Crenate, setae relatively stout, clearly terminating veins	*Allen 5812* (US, holotype)
*H.excelsum* (Ducke) Cuatrec.	Near secondary vein loops toward margin, relatively sparse	1 pair at margin	Crenate, setae present	*Museu Goeldi 9672* (US)
*H.glaziovii* (Urb.) Cuatrec.	Absent	1 pair at margin, nearly abaxial	Serrate, setae present	*Pabst 4726* (US)
H.glazioviivar.angustifolium Cuatrec.	Absent	1 pair at margin, nearly abaxial	Crenate, setae scars	*Glaziou 16724* (US, holotype)
*H.mapiriense* Cuatrec.	Sparse, toward margin	0–2 pairs	Crenate, setae scars	*Buchtien 1518* (US, isotype)
*H.melanocarpum* (Cuatrec.) Cuatrec.	Very rare on few leaves, scattered, hard to distinguish from potential leaf damage	Absent	Crenate, with setae	*Cuatrecasas 19909* (US); best gland evidence is on *Prance 28055* (US)
*H.mussunungense* Cuatrec.	Absent	0–1 pair at margin, nearly abaxial	Crenate, setae present	*Folli 1393* (US, holotype)
*H.obovatum* (Benth.) Cuatrec.	Sparse, scattered	0–1 pair at margin, nearly abaxial	Subentire, setae scars sparse, hirsute	*Schomburgk 1359* (US)
*H.ottohuberi* Cuatrec.	Row near margin	1–3 pairs, oval	Obscurely crenate, setae present	*Steyermark & Bunting 102442* (US, holotype)
*H.piraparanense* Cuatrec.	Tiny, sparse, near margin	1–2 pairs	Crenate, setae scars	*Schultes & Cabrera 15922* (US, holotype); glands better developed on *García-Barriga 14287* (US)
*H.procerum* (Little) Cuatrec.	Towards margin along loops between secondary veins	1(–2) pairs at margin	Crenate, setae scars	*Little & Dixon 21148* (US)
*H.subcrenatum* (Benth.) Cuatrec.	Absent	1 pair at margin	Crenate, setae scars	*Martin s.n.* (US, type fragment)
*H.villosum* (Fróes) Cuatrec.	Tiny, sparse, in row near margin	1 pair at margin, small	Serrate, setae scars	*Humbert & Schultes 27363* (US)
***Hylocarpa***				
*H.heterocarpa* (Ducke) Cuatrec.	Large, erratic, near secondary or intersecondary veins, 1/2–2/3 exmedial distance	Absent	Subentire, setae scars	*Ducke [JBRJ-30137*] (US, isotype
***Sacoglottis***				
*S.amazonica* Mart.	Small, near margin	1 pair	Obscurely crenate, setae scars	*J. Wurdack 293* (US)
*S.ceratocarpa* Ducke	Relatively large, near margin	1 pair, large, narrowly elliptic, at margin of leaf base extension	Subentire, setae present	*Fróes 21192* (US)
*S.cydonioides* Cuatrec.	Tiny, near margin	1 pair, elliptic, sometimes large	Serrate, tooth tips with darkened spots (setae scars?)	*B.W. [Surinam Forestry Dept.] 4720* (US)
*S.gabonensis* (Baill.) Urb.	Absent	1(–2) pairs at margin	Crenate, setae present	*McPherson 13714* (US)
*S.guianensis* Benth.	Small, row near margin	0–1 pair at margin	Shallowly crenate; setae present, more persistent than is typical	*Schomburgk 574* (NY, syntype)
S.guianensisvar.hispidula Cuatrec.	Tiny, near margin	Absent	Serrulate, setae scars	*Krukoff 6653* (US, holotype)
S.guianensisvar.maior Ducke	Small, row near margin	Absent	Crenate; setae more persistent than is typical	*Ducke [JBRJ- 23818* (US, isotype)
*S.holdridgei* Cuatrec.	Small, row near margin	1(–2) pairs	Shallowly crenate, setae scars	*Holdridge 5164* (US, isotype)
*S.maguirei* Cuatrec.	Sparse (0–2), tiny, near leaf apex margin, most leaves lacking	Absent	Shallowly crenate, setae scars	*Maguire et al. 30693* (US, holotype)
*S. mattogrossensis* var. *subintegra* (Ducke) Cuatrec.	Small, sparse, near margin	Absent	Subentire, setae scars obscure	*Ducke [JBRJ-23820*] (US, isotype)
*S.ovicarpa* Cuatrec.	Absent	1 pair	Subentire, setae scars	*Cuatrecasas 17226* (US)
*S.perryi* K. Wurdack & C.E. Zartman	Tiny, near margin	1 pair, relatively small, obscure	Shallowly crenate, setae present	*McDowell 2920* (US)
*S.trichogyna* Cuatrec.	Absent	1 pair	Shallowly crenate, setae scars	*Holdridge 5216* (US, isotype)
***Schistostemon***				
*S.auyantepuiensis* Cuatrec.	Tiny, with reticulate veins near margin	1 pair, large elliptic	Very obscurely crenate, setae scars	*Davidse & Huber 22804* (US)
*S.densiflorus* (Urb.) Cuatrec.	Small, row near margin	0–1 pair	Obscurely crenate, setae present	*Schomburgk 543* (US isotype)
*S.dichotomus* (Urb.) Cuatrec.	Small, row near margin	1 pair	Crenate, setae scars	*Sabatier 989* (US)
*S.fernandezii* Cuatrec.	Near margin	1 pair, oval	Obscurely crenate, small setae present	*Fernandez 2276* (US, holotype)
*S.macrophyllus* (Benth.) Cuatrec.	Row near margin, widely spaced	1 pair, elliptic	Shallow teeth, setae scars	*Ducke 1744* (US)
*S.oblongifolius* (Benth.) Cuatrec.	Near margin	1–2 pairs	Small, shallow teeth, setae scars	*Maas et al. 6804* (US)
*S.reticulatus* (Ducke) Cuatrec.	Near margin	1–2 pairs at base, up to 3 per side near apex	Obscurely crenate	*Ducke [JBRJ- 23819*] (US, isotype), *Klug 1564* (US)
S.reticulatusvar.froesii Cuatrec.	Near margin	1 pair at base, no glands at apex	Subentire, setae scars sparse	*Fróes 21370* (US, isotype)
*S.retusus* (Ducke) Cuatrec.	Near margin, relatively regular all the way to tip	1 pair, large	Subentire, setae scars sparse	*Cuatrecasas 7203* (US)
*S.sylvaticus* Sabatier	Row near margin	1–2 pairs at base	Very obscurely crenate, setae scars	*Loubry 1074* (US)
***Vantanea***				
*V.bahiaensis* Cuatrec.	Tiny, near secondary or intersecondary veins	1 pair	Entire	*Belém & Magalhães 748* (US); *Carvalho & Gatti 484* (NY, holotype)
*V.barbourii* Standl.	Near secondary or intersecondary veins	1 pair	Entire	*Hartshorn 2139* (US)
*V.celativenia* (Standl.) Cuatrec.	Row 1/3 exmedial distance	1 pair, often others near base	Entire	*Krukoff 7182* (US, isotype)
*V.compacta* (Schnizl.) Cuatrec.	Tiny, near intersecondary veins near margin	0–4 pairs per side	Entire	*Hatschbach 21265* (US)
*V.deniseae* W.A. Rodrigues	Near secondary or intersecondary veins	Absent	Entire	*Freitas et al. 860* (US)
*V.depleta* McPherson	Near secondary veins, row half exmedial distance	1 pair	Entire	*McPherson & Stockwell 10892* (US, isotype)
*V.guianensis* Poir.	Scattered	Absent	Entire	*Maguire et al. 56006* (US)
*V.macrocarpa* Ducke	Scattered	Absent	Entire	*Ducke 2230* (US)
*V.magdalenensis* Cuatrec.	Tiny, near secondary and intersecondary veins	1 pair	Entire	*Lamb 133* (US, holotype)
*V.micrantha* Ducke	Scattered	Absent	Entire	*Ducke 751* (US)
*V.minor* Benth.	Large, sparse, proximal half near primary vein	Absent	Entire	*Liesner 23974* (US)
*V.morii* Cuatrec.	Absent	2(–4) pairs	Entire	*Mori & Benton 13181* (US, holotype)
*V.obovata* Benth.	Near secondary or intersecondary veins	1–2 pairs	Entire	*Anderson et al. 36781* (US)
*V.occidentalis* Cuatrec.	Large, near intersecondary veins, row 1/3 exmedial distance	Absent	Entire	*Patiño 12* (US)
*V.ovicarpa* Sabatier	Tiny, close to secondary veins	0–1 pair, rarely others near base	Entire	*Granville 10792* (US)
*V.paraensis* Ducke	Near secondary or intersecondary veins	1 pair, often others along secondary near base	Entire	*Ducke [JBRJ-23430*] (US)
*V.parviflora* Lam.	Along loops of secondary veins, near margin	Row of 2–4(5) pairs, usually close to mid-vein	Entire	*Prance et al. 17770* (US)
V.parvifloravar.puberulifolia Cuatrec.	Along loops of secondary veins, near margin, usually surrounded by vein	2 (1–3) pairs, closer to midvein	Entire	*Ducke [JBRJ-23428*] (US, holotype)
*V.peruviana* J.F. Macbr.	Large, row, mostly near intersecondary veins, 1/4–1/3 exmedial distance	Absent	Entire	*Rimachi 4577* (US)
*V.spichigeri* A.H. Gentry	Row, 1/5 way to margin, often near secondary veins	1–2 pairs	Entire	*Abadie s.n. 12-9-74* (US), *Spichiger et al. 1743* (NY, isotype)
*V.spiritu-sancti* (Cuatrec.) K. Wurdack & C.E. Zartman	Sparse, small along secondary or intersecondary veins, half exmedial distance	1–2 (type); 5–7 pairs along proximal edge	Entire	*Dolcolmo [JBRJ-86212*] (US, isotype fragment); *Silva et al. 1436* (US), more abundant
*V.tuberculata* Ducke	Along secondary veins, half exmedial distance	Absent	Entire	*Ducke [JBRJ-30134*] (US, isotype)
